# Natural Combatants: How Herbal Monomers Regulate Autophagy in Ovarian Cancer

**DOI:** 10.2174/0118715206401652251016063228

**Published:** 2026-02-13

**Authors:** Mengyao Gao, Yufei Zhu, Yang Yu, Danni Ding, Yu Wang, Fengjuan Han

**Affiliations:** 1 Department of Obstetrics and Gynecology, Heilongjiang University of Chinese Medicine, Harbin, Heilongjiang 150040, China;; 2 Department of Obstetrics and Gynecology, The First Affiliated Hospital of Heilongjiang University of Chinese Medicine, Harbin, Heilongjiang 150040, China

**Keywords:** Ovarian cancer, autophagy, traditional Chinese medicine monomers, gynecological malignancy, chemotherapy sensitivity

## Abstract

**Introduction:**

Ovarian cancer (OC) is a lethal gynecological malignancy, with current therapies constrained by drug resistance and side effects. Autophagy plays a dual role in OC, while Traditional Chinese Medicine (TCM) monomers, as natural bioactive compounds, demonstrate significant potential in regulating autophagy and combating tumors. This review aims to summarize the mechanisms by which TCM monomers regulate autophagy in OC, providing a theoretical basis for TCM-based drug development and clinical applications.

**Methods:**

Relevant literature was retrieved from databases including PubMed, Web of Science, and CNKI. The action targets and signaling pathways of TCM monomers were summarized to elucidate their mechanisms in regulating OC autophagy.

**Results:**

TCM monomers (*e.g.*, ginsenoside Rg3, curcumin) bidirectionally regulate OC autophagy through pathways such as PI3K/AKT/mTOR and MAPK; some enhance chemotherapy sensitivity by inducing excessive autophagy or inhibiting protective autophagy.

**Discussion:**

TCM monotherapy offers unique advantages in the treatment of OC through precise regulation of autophagy. However, most studies are limited to *in vitro* experiments, and there is insufficient *in vivo* efficacy and clinical translational evidence.

**Conclusion:**

Validating the complex action network of TCM monomers through multi-omics and clinical studies, and exploring their synergistic effects with conventional chemotherapy, is crucial for advancing the development of natural anti-ovarian cancer drugs.

## 1 INTRODUCTION

According to the global cancer data in 2020, OC is the eighth most common of all new cancer cases in women, accounting for an estimated 3.7% of cases and 4.7% of cancer deaths in 2020, and OC has the fourth highest incidence and the third highest mortality rate among women's tumors [1, 2]. Because the disease is located in the pelvic cavity, the early clinical symptoms are mild. There is a lack of accurate noninvasive early screening methods, resulting in more than 70% of patients being diagnosed at a late stage, with a 5-year survival rate of less than 45% [3, 4]. At present, the treatment of OC is mainly tumor cytoreductive surgery combined with platinum-based chemotherapy. Under this treatment, the consequences of multidrug resistance are easy to occur, resulting in poor efficacy of OC. Recurrence and metastasis are also great challenges facing the clinic [5]. Therefore, it is urgent to explore new anti-cancer therapies, and solving the problem of multidrug resistance is the key. Autophagy is a dynamic process that maintains the normal metabolism and homeostasis of cells through their “self-digestion” [6]. It has a complex and close relationship with tumors, and the challenge of seeking new solutions to multidrug resistance may be broken through autophagy. Numerous studies have confirmed that herbal monomers modulate cellular autophagy to treat tumors with significant advantages, and modern studies have shown that autophagy is one of the important mechanisms as a natural medicine to play an anticancer role, in which the mechanism is mostly by reversing the resistance of cancer cells to drugs or improving their sensitivity or can enhance the regulation of the cancer cell death, reduce proliferation and migration and invasion [7-10], and OC is no exception. Perhaps digging deeper into this mechanism could take OC treatment to the next level.

Natural medicines possess a greater variety of structures and biological activities compared with conventional therapeutic drugs and have gradually become a tremendous resource bank for the research and development of anti-cancer drugs [11]. The TCM monomer is a substance with a clear chemical structure, a relatively single component, and unique biological activity that is extracted and separated from TCM. It is an important material basis for the efficacy of TCM [12]. In recent years, as our understanding of the anti-cancer activity of TCM monomers has deepened, the clinical efficacy of various forms of TCM in treating OC has gradually been recognized. Moreover, several *in vivo* and *in vitro* experiments have confirmed that TCM monomers can affect the activity and drug sensitivity of OC cells by regulating autophagy, indicating a feasible path for finding new anti-cancer chemical drugs in natural medicines, and it may become a conventional supplementary means after the current Western medicine treatment for OC. Regrettably, research on the regulation of autophagic activity in OC by different TCM monomers is limited to *in vivo* cell experiments and *in vitro* animal experiments. The underlying potential mechanisms of their effects have not been clearly elucidated, and it has not yet advanced to the clinical trial stage. Therefore, the safety and efficacy of these drugs need to be further verified through additional studies. Thus, this article summarizes the mechanism by which numerous natural TCM monomers affect OC by regulating autophagy, hoping to attract more scholars to engage in further studies to clarify the mechanism and advance to the clinical trial stage. This is of great significance for developing new anti-cancer drugs, resolving the problem of drug resistance in cancer cells, and contributing to the early clinical application of new drugs.

## 2 SEARCH STRATEGY AND SELECTION CRITERIA

**Fig. (1):**
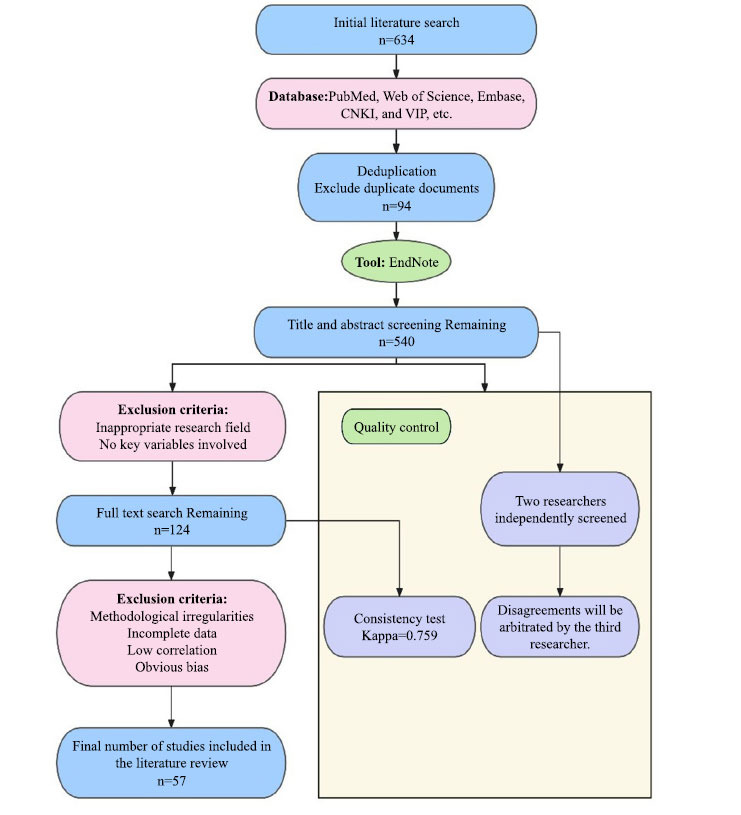
Literature screening flowchart.

We used “autophagy”, “ovarian cancer”, and related terms as keywords in PubMed, Web of Science, Embase, CNKI, and VIP databases, and relevant literature published between 2014 and 2024 was analyzed. The literature screening process involved three stages: initially, 634 articles were retrieved using keywords; after removing 94 duplicate articles using EndNote, 540 articles remained. Next, two researchers screened the titles and abstracts based on criteria such as relevance to the research topic, excluding 416 articles and leaving 124. Finally, full-text articles were carefully reviewed, and 67 were excluded based on criteria such as inadequate study design, incomplete data, and low relevance to the core research question, resulting in 57 articles being included. The Kappa value for the screening process was 0.759, with discrepancies resolved by a third researcher, indicating high reliability. The criteria for excluding biased literature included methodological issues such as sample selection bias, selective reporting of results, significant conflicts of interest that could affect objectivity and were not controlled for bias, and articles that were merely opinion-based without empirical support. Through a rigorous screening process and bias exclusion criteria, the relevance, scientific rigor, and objectivity of the included literature were ensured, and the articles focused on animal and cellular experiments (Fig. **1**).

## 3 AUTOPHAGY AND THE OCCURRENCE OF OC

The concept of “autophagy” first emerged in 1962, when scientists Ashford *et al*. put forward the term to characterize this intracellular phenomenon [13]. As a conserved intracellular metabolic process in mammals, autophagy encompasses three forms: macroautophagy (the focus here, also referred to as type II programmed cell death), microautophagy, and chaperone-mediated autophagy [14]. Macroautophagy works through phagophores that enclose damaged organelles and biomacromolecules, forming autophagosomes. These then merge with lysosomes to create autolysosomes, where acidic hydrolases break down contents for recycling into amino acids, fatty acids, and nucleotides—a process vital for maintaining cellular homeostasis [15]. Autophagy functions as a double-edged sword in systemic diseases. Under normal conditions, it eliminates aged organelles and metabolites. When cells face stress, heightened autophagy protects them by clearing debris, yet overactivation can lead to autophagic cell death, a process distinct from apoptosis [16]. In tumors, autophagy’s role in regulating apoptosis varies with cellular context: it hinders early carcinogenesis by inducing apoptosis but supports the survival, growth, and chemoresistance of established tumors [17]. Previous studies have shown that autophagy primarily provides the energy necessary for tumor survival during the later stages of tumor growth. However, certain Chinese herbal compounds may inhibit autophagy during this stage [18], cutting off the energy supply to tumor cells and killing them. Recent studies have revealed that, after tumor cells form, certain novel chemotherapy drugs (such as Olaparib) can activate the autophagy signaling pathway in OC cells [19]. Overly activated autophagy disrupts mitochondrial integrity, leading to the increased release of apoptosis-related factors, such as cytochrome C. This triggers the caspase cascade reaction, ultimately inducing cell apoptosis. This dual nature is also observed in OC [20, 21], making autophagy modulation a promising therapeutic avenue.

Autophagy represents a complex programmed death mechanism, involving multiple signaling pathways and regulatory factors that form an elaborate interaction network. This study emphasizes how individual Chinese herbal compounds influence autophagy either directly or indirectly through specific signaling pathways or targeted factors, thus offering new therapeutic strategies for OC treatment.

**Fig. (2):**
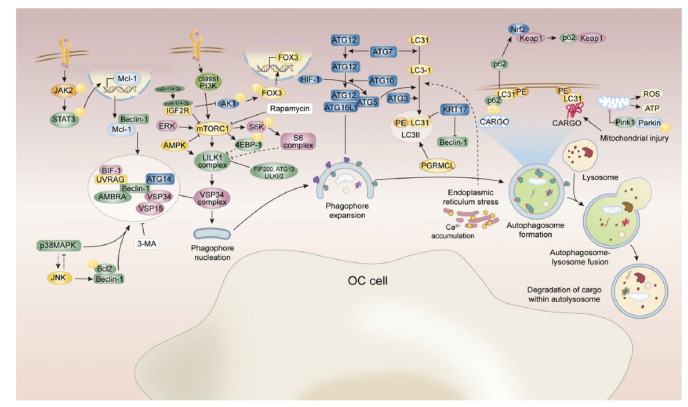
The autophagy regulation diagram in OC.

The PI3K/AKT pathway plays a key role: external stimuli activate PI3K, leading to Akt phosphorylation, which in turn promotes OC cell survival and proliferation. Inhibiting this pathway switches on autophagy, while phosphorylated Akt suppresses autophagy-related genes by phosphorylating FOXO3 [22, 23]. Along similar lines, microRNA-144-3P targets IGF2R, lowering its expression and blocking Akt/mTORC1 phosphorylation to boost autophagy [24]. The mTOR pathway, made up of mTORC1 and mTORC2, controls autophagy: mTORC1 activates p70S6K, and when inhibited, it triggers autophagy [25, 26]. ERK strengthens mTORC1 activity, whereas AMPK inhibits it and aids in forming the ULK1 complex [27]. Within the tumor microenvironment, cytokine stimulation sets off JAK/STAT3 signaling. Phosphorylated STAT3 moves into the nucleus, encouraging Mcl-1 transcription; Mcl-1 then binds to Beclin-1, inhibiting autophagy [28, 29]. Beclin-1, a central inducer of autophagy, forms the Vps34 complex [8]. The P38 MAPK/JNK pathway phosphorylates Bcl-2, weakening its attachment to Beclin-1 and starting autophagosome formation [30]. After the double membrane assembles, ATG proteins draw in the ATG5-ATG12-ATG16L1 complex and LC3-II to phagophores. LC3-I undergoes lipidation by ATG7/ATG3 to become LC3-II, a marker for autophagosome maturation. ATG5 promotes LC3-II formation, with HIF-1 enhancing ATG5’s function [31]. PGRMC-1 and endoplasmic reticulum stress-induced calcium accumulation also aid in LC3-II generation, while KRT17 and Beclin-1 work against it [32, 33]. Ubiquitinated cargo gets enclosed in autophagosomes through the interaction between P62 and LC3-II. P62 levels are inversely related to autophagic activity, as it gets degraded in autolysosomes [34]. P62 adjusts the binding between Nrf2 and Keap1 to regulate degradation. Mitophagy (*e.g.*, *via* the Pink1/Parkin pathway) and endoplasmic reticulum stress-induced autophagy impact cancer cell behavior and chemosensitivity [35, 36]. The Pink1/Parkin pathway mediates mitophagy: Pink1 phosphorylates Parkin, allowing LC3-II to bind to cargo and clear damaged mitochondria [37]. Finally, the fusion of autophagosomes and lysosomes completes autophagy in OC (Fig. **2**).

In conclusion, autophagy’s involvement in OC progression underscores its potential as a therapeutic target to enhance clinical outcomes.

## 4 PHYTO-POWER: UNLOCKING AUTOPHAGY'S POTENTIAL IN OC WITH HERBAL MONOMERS

Chinese herbal monomers have become the main object of studying the TCM treasury. They have broken through the bottleneck of insufficient bioavailability of TCM. With the purity of their components and the clarity of their curative effects, they provide precise intervention methods for the anti-cancer field. Especially in the treatment of OC, TCM monomers not only play an important role in alleviating the clinical symptoms of patients, reducing the development of drug resistance, and preventing disease recurrence, but also the autophagy phenomenon in cancer cells is more in line with the unique concept of yin-yang balance in TCM, showing its unique therapeutic potential [38]. Modern experiments have shown that most TCM monomers, acting as natural autophagy inhibitors or inducers in OC treatment, can not only play an independent role but also be combined with existing anti-cancer drugs to form a multi-faceted treatment strategy. The mechanism involves both promoting the occurrence of death-induced autophagy in OC cells and inhibiting protective autophagy [39, 40]. However, it should be noted that some active components of TCM can also promote the late-stage protective autophagy of OC [41]. This process can be verified by the combined use of autophagy-specific inhibitors (including the early autophagy inhibitor 3-Methyladenine (3-MA), the late autophagy inhibitor Chloroquine (CQ), Bafilomycin A1 (BafA1)), the autophagy activator Rapamycin (RAPA), the mTOR activator MHY1485, the JAK2 inhibitor AG490, the JAK2 activator Interleukin-6(IL-6), the antioxidant N-Acetylcysteine(NAC), etc., and even some combinations of these can produce a synergistic effect [42]. Among them, 3-MA and CQ are currently classic autophagy inhibitors used to study autophagy. 3-MA inhibits the formation of autophagosomes by inhibiting the activity of type III PI3K, a key enzyme for autophagy initiation [43]. CQ inhibits the combination of lysosomes and autophagosomes, thereby preventing the autophagosome from degrading LC3-II [44]. In summary, this paper primarily categorizes and organizes the chemical structures, providing a detailed explanation of how they can balance inducing cancer cell death with enhancing cancer cell survival. By exploring this delicate balance, the intrinsic mystery of treating OC can perhaps be unraveled. This could provide a new theoretical basis and practical direction for the TCM treatment of OC, improve sensitivity to drug treatment, and further promote the in-depth development of research in this field.

### 4.1 Harnessing Herbal Power: Inducing Fatal Autophagy in OC Cells

Many active monomers of TCM can inhibit the apoptosis, growth, and metastasis of OC by triggering lethal autophagy. The current research is mainly divided into the following six types of compounds. This process is primarily achieved by inhibiting different signal pathways, such as inhibiting the PI3K/AKT/mTOR pathway, the mTOR/S6K pathway, or the JAK2/STAT3 pathway. It can also induce autophagy by increasing autophagy-related proteins such as LC3-II and Beclin-1 and reducing autophagy-related markers such as P62, thereby inhibiting the growth of cancer cells and promoting their apoptosis. Monomers with this biological activity may serve as a new therapeutic approach, opening up a new avenue for slowing the progression of OC and addressing the problem of chemotherapy resistance.

#### 4.1.1 Natural Terpenoids

Natural terpene compounds such as pseudolaric acid B, Poria acid A, and triptolide, which are extracted from TCM, possess significant antitumor activity. They can induce lethal autophagy in OC by targeting key signaling pathways such as PI3K/AKT/mTOR and JAK2/STAT3, by inhibiting the expression of related autophagy proteins, or by suppressing autophagic flux, thereby inhibiting the progression and metastasis of OC.

Pseudolaric acid B is a diterpenoid compound extracted from golden larch bark. It can exert an anti-tumor effect by promoting cancer cell apoptosis and autophagy and blocking the cancer cell cycle [45]. It is likely to cause the levels of p-AKT and p-mTOR to decrease in a drug concentration-time-dependent manner through the PI3K/AKT/mTOR pathway and upregulate the expressions of LC3-II and Beclin-1, thereby inducing autophagy in OC cells and achieving the goal of effectively inhibiting the viability of HO8910 cells [46]. Poria acid A is a triterpenoid compound derived from Poria cocos [47]. Data indicate that it induces apoptosis and autophagy in OC cells by regulating the mTOR/S6K signal transduction axis, thereby inhibiting the occurrence and development of cancer cells [48]. Triptolide has various pharmacological effects, including anti-tumor and immune regulation [49]. *In vitro* and *in vivo* experiments have shown that the mechanism of its induction of autophagy in SKOV3/DDP cells is related to the inhibition of the activated JAK2/STAT3 pathway. It downregulates the expression of Mcl-1 and reduces the formation of the Beclin1/Mcl-1 complex, thereby enhancing the autophagic flux mediated by Beclin-1. This process inhibits cell proliferation, induces the growth of SKOV3/DDP OC xenograft tumors, and enhances the sensitivity of DDP *in vivo* [50, 51]. Salidroside is regarded as one of the main functional components of Rhodiola rosea [52]. It can inhibit cell proliferation and induce apoptosis and autophagy, and its synergistic effect when combined with paclitaxel may be through down-regulating the expression of KRT17, thereby enhancing the sensitivity of paclitaxel to SKOV3 cells [33]. The ability of the aforementioned active monomers to inhibit the activity of OC cells and induce apoptosis can be reversed by autophagy inhibitors. However, while reducing autophagy activity, the apoptosis level is also decreased, indicating that these compounds exert a synergistic anti-cancer effect through autophagy and apoptosis (Table **1**).

In brief, the above-mentioned monomers have become potent regulators of cell signals. These findings emphasize the significance of exploring natural products for cancer treatment and highlight the necessity of further studies to reveal their mechanism of action and optimize their therapeutic potential.

#### 4.1.2 Alkaloids

However, alkaloids such as cepharanthine and tetrandrine can enhance the autophagic activity of OC by regulating key molecules in classic autophagy signaling pathways, such as PI3K/AKT/mTOR and MAPK/JNK, or by influencing related proteins, including those involved in mitochondrial autophagy. Interestingly, some monomers can even be used in combination with autophagy inhibitors to enhance their inhibitory effect on cancer cells.

Cepharanthine is a natural active alkaloid derived from the medicinal plant Stephania japonica. It has garnered much attention because it can inhibit the proliferation of tumor cells, induce autophagy of tumor cells, and reverse chemotherapy resistance [53]. It should be noted that, on the one hand, it may induce autophagy activation in SKOV3 cells and reduce their survival rate by inhibiting the PI3K/AKT/mTOR pathway. The suitable concentrations are 8 and 16 µmol/L. On the other hand, when it is used in combination with 3-MA, autophagy is inhibited, and SKOV3 cells lose the ability to alleviate the stress induced by it through autophagy. This may lead to an increase in cell death because 3-MA blocks the late-stage self-protection mechanism of the cells, enhancing the cytotoxicity of the drug, thereby strengthening its inhibitory ability on the viability of cancer cells [54]. And this effect is also manifested in other TCM components such as tetrandrine. Tetrandrine is extracted from Stephania tetrandra and has become a commonly used adjuvant therapeutic drug for tumors in clinical practice [55]. It is found that tetrandrine at concentrations of 4 and 8 µmol/L can maximally upregulate the number of autolysosomes and the expression of LC3-II and P62 proteins in OC by inhibiting the PI3K/AKT/mTOR signaling pathway. The transient increase in the P62 level may be related to enhanced upstream expression, leading to aggregation in a short period that has not been properly degraded [56, 57]. The matrine derived from Sophora flavescens can promote autophagy in OC cells by inhibiting the phosphorylation of the Akt/mTOR signaling pathway, and autophagy and apoptosis act synergistically. It induces a significant increase in the percentage of G0/G1 phase cells in a dose-dependent manner, showing a great advantage in overcoming chemotherapy drug resistance and reducing toxic side effects [58]. Excitingly, its effect on autophagy is throughout the entire process. Whether it is the induction of early autophagic death or the inhibition of late protective autophagy, these processes can exert their biological activity, thereby significantly enhancing the sensitivity of cancer cells to radiotherapy and chemotherapy [59]. Harmine is mainly derived from β-carboline alkaloids in the medicinal plant Peganum harmala L [60]. *In vitro* and *in vivo* experiments proved that it not only promoted autophagy by regulating PI3K/AKT/mTOR/FOXO3 signaling pathway, but also played its anti-OC role by promoting autophagy-dependent iron death, and pyroptosis, in which the former two were involved [22]. Neferine is a bis-benzylisoquinoline alkaloid derived from the Lotus Plumule [61]. In 2016, a study demonstrated that neferine induces autophagy in human OC cells through the p38 MAPK/JNK pathway, and it can also induce G1 cell cycle arrest and apoptosis [62]. The main components of Dictamnus dasycarpus Turcz include dictamnine, which can act on OC cells through multiple targets and multiple pathways [63]. KEAP1 may be the key target for the treatment of OC with dictamnine. By reducing its expression, it can induce autophagy in cells, and this substance may inhibit cell proliferation and induce cell apoptosis through the PI3K pathway [64]. 19-Hydroxytelocinobufagin is one of the active components extracted from the dry secretions of toad species of the Bufonidae family [65]. *In vitro* studies have demonstrated that it can promote the expression of Beclin-1, Atg5, LC3 II, Pink1, and Parkin, and inhibit the expression of P62. This indicates that it induces mitochondrial autophagy in a concentration-dependent manner, induces OC cell apoptosis, and inhibits escape. The specific mechanism is that, in an *in vitro* environment, this monomer increases ROS levels through the mitochondrial pathway and enhances Parkin recruitment in the mitochondria. Compared with Chinese herbs themselves, it eliminates the toxic effects, and it can also induce OC cells to undergo G2/M phase cycle arrest [66]. The expression levels of ATG5 and LC3-II are also affected by the active component of TCM, lycorine, which comes from the Lycoris genus [67]. It can significantly upregulate the expression of the aforementioned proteins and downregulate the expression levels of Bcl-2, p-AKT, and P62. These results indicate that lycorine may activate apoptosis and autophagy through the Bcl-2 and PI3K/AKT pathways, thereby inhibiting tumor growth [68]. Berberine is derived from the Chinese herb Coptis chinensis [69]. Among them, berberine (200μmol/L) significantly increased the expression of endoplasmic reticulum stress-related proteins (GRP78 and CHOP) and autophagy-related proteins, indicating that it may promote ER stress in SKOV3 cells by regulating cell autophagy. This finding provides a new idea for the treatment of OC at the organelle level [70, 71] (Table **2**).

In simple terms, suppressing autophagy can block the regulatory signaling pathways mediated by alkaloids as mentioned above, thereby inducing the anticancer process of autophagy. They have fewer toxic side effects within the effective dose range, higher selectivity, and possess certain therapeutic potential, providing more options for the clinical treatment of OC.

#### 4.1.3 Natural Dietary Polyphenols

Natural dietary polyphenols derived from TCM, such as kaempferol and curcumin, also contribute to the regulation of autophagy, apoptosis, oxidative stress, and aging in the body. Kaempferol is a kind of polyphenolic compound derived from TCM such as Kaempferia galanga and Scutellaria barbata [72]. After treating OC cells with it, the Ca^2+^ level is increased, and the endoplasmic reticulum stress-mediated cytotoxic autophagy is activated. The expressions of LC3II, Beclin-1, and ATG5 are significantly enhanced [73]. Resveratrol is a non-flavonoid polyphenolic compound extracted from Veratrum [74]. Current studies indicate that resveratrol induces autophagic cell death and promotes apoptosis in OC cells through two pathways. On the one hand, the experimental study by Wang *et al* indicates that after treatment with a resveratrol concentration of 25 μmol/L for 24 hours, the expression levels of LC3 and Beclin-1 in SKOV3 cells increase, while the protein levels significantly decrease after the addition of 3-MA [75]. On the other hand, it can function by inducing the generation of ROS and oxidative stress [76]. Protocatechuic acid can be found in *Suberect spatholobus*. Studies have proved that it can be developed as a potential chemotherapeutic drug for human OC [77]. After treatment with protocatechuic acid, it can induce cell cycle arrest at the G2/M phase, leading to an upregulation of LC3-II expression and the induction of GFP-LC3 punctate formation. When combined with an autophagy inhibitor for treatment, it can reduce the cytotoxicity produced by protocatechuic acid targeting OVCAR-3 cells, indicating that it exerts a synergistic effect on OC cells through autophagy and apoptosis [78]. Ellagic acid is a natural polyphenolic active component derived from Chinese medicinal herbs such as Euphorbia and gallnuts [79]. It activates cytotoxic autophagy by inhibiting the mTORC1/Akt pathway.

Additionally, it can activate AMPK to inhibit the growth, migration, and invasion of SKOV3. The specific mechanism is to increase the levels of Beclin-1, ATG-5, and LC3I/II, and to reduce the levels of P62 and Bcl-2 [80] (Table **3**). These polyphenolic compounds induce autophagy through different mechanisms to exert an impact on the growth and progression of OC, demonstrating their potential in cancer treatment.

#### 4.1.4 Saponins

Compounds such as Ginsenoside 20 (S)-Rg3 and Polyphyllin I can induce autophagy in OC in a dose-dependent manner, thereby exerting an anti-tumor effect. This may make these monomers more definite anti-tumor drugs as a new direction for clinical application.

Ginsenoside 20 (S)-Rg3 is a widely used ginseng extract [81]. Studies have shown that it can induce autophagy in OC cells by upregulating ATG5 and ATG7, thereby inhibiting the migration and invasion capabilities of these cells, and thus becoming a potential anti-OC strategy [82]. Treating OC cells with Eclalbasaponin II derived from Eclipta prostrata can act on the JNK, p38, and mTOR signaling pathways in a dose-time-dependent manner. This treatment significantly increases the expression of LC3-II and Beclin-1 and reduces the level of P62, inducing autophagy and thereby causing cell apoptosis [83, 84]. Macranthoside B is a saponin compound in Lonicera macranthoides that can block cell proliferation and induce cell death in several cancer cells [85]. Macranthoside B can increase LC3-II expression, induce cytotoxic autophagy in A2780 cells, and regulate the expression of upstream genes involved in cell apoptosis. The realization of this ability may induce cell apoptosis through the ROS/AMPK/mTOR pathway [86]. Polyphyllin I is derived from the TCM Paris polyphylla [87]. Interestingly, Polyphyllin I can inhibit cell death when 3-MA is added instead of HCQ, confirming that it causes cell death by initiating early autophagy, which leads to an increase in autophagic flux [88]. More interestingly, another study has proved that Polyphyllin I can not only induce early fatal autophagy but also inhibit the late autophagy of Skov3 cells by increasing the levels of LC3-II, LC3-I/LC3-II, and P62, thereby playing a continuous role throughout the entire process of cell death [89]. Saikosaponins are saponin compounds derived from the TCM Bupleurum [90]. It can reduce the LC3-I/LC3-II ratio in SKOV3 cells, increase the expression of Beclin-1, enhance autophagy intensity, and lead to cell apoptosis, thereby inhibiting the growth of SKOV3 cells. Beclin-1/Bcl-2 is a definite molecular connection between autophagy and apoptosis. When the expression level of Beclin-1 increases, the pro-apoptotic proteins BAK/BAX can be released from their combination with Bcl-2, thereby promoting cell apoptosis. This may be one of the reasons why Saikosaponins promote autophagy and apoptosis in SKOV3 cells [91]. Due to the reduced therapeutic effect on OC in the later stage caused by DDP resistance, Astragaloside II derived from Astragalus membranaceus may induce autophagy by inhibiting the AKT/mTOR signaling pathway. Its characteristics are the up-regulation of LC3II expression and the down-regulation of P62 expression, thereby increasing the sensitivity of DDP in the treatment of OC [92] (Table **4**).

Saponin compounds can enhance the apoptosis of OC and inhibit its progression by targeting related signaling pathways and modulating autophagy proteins. They can also play a role in inhibiting cancer in synergy with chemotherapy drugs.

#### 4.1.5 Flavonoids

Some other flavonoid compounds can also prevent the occurrence and development of OC by regulating autophagy. Icariside II is a kind of flavonoid natural compound extracted from the TCM Epimedium [93]. When SKOV3 cells are treated with Icariside II at a concentration of 60μmol/L, it inhibits the expression of IGF2R, Akt, mTOR, and P62, promotes the expression of p-AMPK, Beclin-1, and ATG-5, and upregulates the level of miR-144-3p to target IGF2R. This indicates that Icariside II can induce autophagy by regulating the AKT/AMPK/mTOR signaling pathway and the miR-144-3p/IGF2R axis [94]. Cardamomin [95], which is mainly derived from the seeds of Alpinia katsumadai, inhibits glycolysis in SKOV3 cells and participates in the promotion of autophagy through the AMPK/S6K1/mTOR-mediated signaling pathway, facilitating the formation and degradation processes of autophagosomes [96, 97]. Diosmetin, a flavonoid compound extracted from liver-soothing and mass-dispersing plants such as Bergamot and Chrysanthemum [98], promotes the conversion of LC3-I to LC3-II and downregulates the expression level of P62, suggesting that it may induce autophagy in OC cells [99]. In addition, nobiletin, a flavonoid compound extracted from *Fructus aurantii*, promotes the formation and continuous accumulation of autophagosomes and mitophagosomes in OC cells by regulating ROS. It activates the expression of LC3 and P62, which results in decreased autophagic flux, inducing lethal autophagy and subsequently leading to pyroptosis in cancer cells. Moreover, the two processes complement each other and jointly exert an anti-cancer effect [100]. Another study indicates that it can inhibit the autophagic degradation of drug-resistant cancer cells by overexpressing the AKT pathway and enhancing cell apoptosis [101]. There is also Garcinone E derived from *Garcinia oblongifolia* [102]. It might enhance the expression levels of LC3-II and P62, block the fusion process of autophagosomes with lysosomes, promote the accumulation of autophagosomes and lysosomes, cause the impairment of autophagic function in OC cells, and thereby induce the occurrence of lethal autophagy [103]. Trichosanthin is derived from Radix Trichosanthis [104]. It upregulates the expression of ATG5, increases autophagic vacuoles, and promotes the cleavage of LC3, indicating that trichosanthin plays a role in human OC by inducing apoptosis and lethal autophagy [105]. Isoliquiritigenin, a natural flavonoid isolated from the roots of *Glycyrrhiza uralensis* [106], can induce cell arrest at the G2/M phase and upregulate the expression levels of LC3-II and Beclin-1, demonstrating that Isoliquiritigenin triggers autophagic cell death in OVCAR5 cells and significantly inhibits the viability of cancer cells [107]. Myricetin is derived from the natural medicine *Myrica rubra* [108]. It can increase the LC3-II/LC3-I ratio in a linear dose-dependent manner, suggesting that autophagy occurs after myricetin intervention in SKOV3 cells [109]. Hyperoside is a flavonoid compound extracted from plants of Hypericaceae [110]. In the experiment by ZHU *et al*. on the effects of hyperoside on the viability, apoptosis, and autophagy of OC cells, it can inhibit their viability and induce autophagic death and apoptosis. This process is partially dependent on autophagy through the PGRMC1/Akt pathway, thereby inhibiting the proliferation of OC cells. It can also make OC cells sensitive to DDP treatment [32]. Baicalin is an effective flavonoid component derived from Scutellaria baicalensis [111]. It can induce death-associated autophagy and apoptosis in OC cells. When combined with cisplatin, it upregulates the expression of Atg5 and Atg12, induces Beclin-1-independent autophagy leading to cell death, and may have the potential to be a chemotherapeutic drug for cisplatin-resistant cancer cells [112]. Luteolin, a flavonoid compound extracted from bamboo leaves [113]. It can induce apoptosis and lethal autophagy in A2780 cells through the AKT-mTOR signaling pathway [114]. Moreover, it can inhibit the expression of the autophagy key enzyme PARP1 induced by cisplatin, thereby suppressing the cisplatin-induced protective autophagy. Additionally, it enhances cisplatin-induced apoptosis and increases the sensitivity of cisplatin [115] (Table **5**).

These natural compounds can regulate autophagy and affect the proliferation of tumor cells. Through various signaling pathways and molecular mechanisms, such as the AKT/AMPK/mTOR signaling pathway and the miR-144-3p/IGF2R axis, they promote autophagy, inhibit the proliferation of OC cells, and induce apoptosis.

#### 4.1.6 Quinones

Quinone compounds such as tanshinone and juglone, active components of TCM, regulate not only some of the aforementioned traditional pathways but also Keap1/Nrf2 and ERK/mTOR, affecting the autophagic activity of OC cells. This process inhibits the growth and proliferation of tumor cells and promotes apoptosis. These components activate the autophagy process by enhancing the expression of autophagy-related proteins and reducing the level of P62. Meanwhile, when combined with drugs such as 3-MA, they can further enhance the inhibitory effect on the proliferation of tumor cells.

Among them, tanshinone is an effective active component of TCM extracted from *Salvia miltiorrhiza*, and the regulation of OC autophagic activity by many of its components deserves attention [116]. For instance, studies have shown that Tanshinone IIA is one of the extracts of *Salvia miltiorrhiza*. When inducing autophagy, it increases the expression level of Beclin-1 and the LC3-II/LC3-I ratio in a drug concentration-dependent manner, thereby inhibiting the proliferation of cancer cells and promoting apoptosis [117]. Juglone is extracted from the green peel of the TCM Juglans regia and has multiple biological effects, such as antibacterial, antioxidant, and anti-tumor [118]. It may promote the autophagy process of SKOV3 cells by inhibiting the AKT/mTOR signaling pathway. This action triggers the upregulation of Beclin-1, ATG7, and LC3-II, and the downregulation of P62 levels, thereby inhibiting the proliferation ability of cancer cells. It can also activate apoptotic factors such as caspase 3 through autophagy, leading to the apoptosis of tumor cells [119]. Damanacanthal is an effective active component extracted from the TCM Rubiaceae [120]. It inhibits the ERK/mTOR signaling pathway, leading to the accumulation of autophagosomes, an increase in the protein level of LC3II, and a decrease in the protein level of P62, thereby inducing autophagy and inhibiting the development of OC cells. Moreover, its effect is comparable to that of cisplatin, and it can synergize with cisplatin to reduce the drug resistance of cancer cells [121] (Table **6**).

In conclusion, inducing autophagic death plays an important role in the inhibition of the malignant biological behaviors of OC by some active components of TCM.

#### 4.1.7 Others

Muscone is a macrocyclic ketone compound [122]. Previous *in vivo* and *in vitro* experiments have found that it can inhibit SHH signal-induced autophagy [123, 124]. Pinoresinol is widely present in various medicinal materials, such as *Forsythia suspensa* and Eucommia ulmoides [125]. *In vivo* and *in vitro* experiments show that it can induce autophagic cell death by up-regulating the expression of LC3-II and Beclin-1 and down-regulating the expression of P62. It may also reduce the mitochondrial membrane potential by inducing autophagy [126]. Osthole is a coumarin compound extracted from the TCM *Cnidium monnieri* and *Angelica sinensis* [127]. It can significantly inhibit the growth of OC cells in a dose-dependent manner through mitochondrial-mediated apoptosis and LC3-mediated autophagy [128]. It is concluded that this type of compound can induce autophagy in OC cells by regulating the expression of autophagy-related proteins and signal pathways, thereby inhibiting the proliferation, migration, and invasion abilities of cancer cells (Table **7**).

These compounds significantly affect the autophagic activity of OC cells by regulating autophagy-related signaling pathways and protein expression, playing a significant role in inhibiting the growth of OC cells. These findings emphasize the importance of exploring natural products for cancer treatment and highlight the need for further studies to reveal their mechanisms of action and optimize their therapeutic potential.

### 4.2 Unleashing Botanical Potential: Triggering Protective Autophagy to Fuel OC Growth

It should be noted that everything has two sides. Many naturally active components of TCM have a dual regulatory effect. They can induce early lethal autophagy in OC cells through multiple pathways and targets, as well as late protective autophagy in cancer cells to a certain extent, thereby protecting them from death by delaying apoptosis. This may pose a new challenge when using TCM to treat OC in clinical practice. However, we do not need to be overly worried, as these compounds have a significant role in promoting tumor cell apoptosis and inhibiting cancer cell growth. The late protective autophagy induced by them may be reversed by the combined use of autophagy inhibitors (such as CQ), which may overcome the limitations of current therapies in the treatment of OC. Therefore, we need to grasp the precise dose and the time point of use. The biological effects exerted by the regulation of the autophagic activity of these compounds are not the same as the substances mentioned above.

Curcumin is a polyphenolic substance extracted from the dried rhizome of turmeric [129], and paeonol is one of the chemical components of the TCM Moutan Cortex and is a kind of monoterpenoid compound [130]. As natural anti-tumor drugs, they can promote cancer cell apoptosis on their own. However, both can induce protective autophagy in OC cells by inhibiting the AKT/mTOR pathway, which may weaken their anti-tumor effects. This process can be reversed by autophagy-specific inhibitors (such as CQ), thereby significantly enhancing the induced apoptosis. Moreover, the combination of curcumin and carboplatin can regulate autophagy by inhibiting the AKT/mTOR signaling pathway, thereby improving the drug sensitivity of cancer cells [131-133]. The alkaloid compound Nitidine Chloride is isolated from the roots of the Zanthoxylum nitidum in the same manner [134]. It can inhibit the expression and phosphorylation of AKT and mTOR proteins, thereby affecting autophagy in OC cells and promoting apoptosis. Furthermore, when used in combination with the autophagy inhibitor CQ, it can increase the sensitivity of OC cells, thereby enhancing their ability to kill cancer cells [135]. Chrysin, mainly derived from the seeds and stem bark of Oroxylum indicum (L.) Vent. of the Bignoniaceae family has an anti-cancer effect similar to the previous monomers [136]. When SKOV3 cells are treated for 24 hours, more autophagosomes appear, increasing the autophagic flux. The combination with CQ also confirms that it induces protective autophagy. At the same time, it also enhances its ability to promote apoptosis and inhibit cell proliferation in OC cells [137]. Vernonia amygdalina sesquiterpene lactone is isolated from Vernonia amygdalina [138]. It can inhibit cell proliferation and arrest the cell cycle at the G2/M phase. However, it can induce protective autophagy in OC cells through the p21 protein. This problem can be perfectly solved by combining it with 3-MA. Its mechanism is to reduce cell viability and promote cell apoptosis by inhibiting the increase of LC3. Its side effects are even significantly lower than those of cisplatin [139]. Daphnetin is a coumarin compound mainly found in Daphne plants [140]. *In vivo* and *in vitro* experiments show that it triggers ROS-induced cell death by inhibiting the AMPK/Akt/mTOR pathway and induces cell-protective autophagy. The combination of it with an autophagy inhibitor may be a potential treatment strategy for OC [141]. Baicalein is one of the main compounds isolated from the roots of *Scutellaria baicalensis*. It has various pharmacological effects, such as reducing ROS production and having anti-cancer properties [142]. After treatment with it, the phosphorylation of ERK and AKT is increased, thereby inducing protective autophagy. However, the combination of CQ and baicalein can significantly reverse the adverse effects of protective autophagy induced by baicalein in these cells [143]. Quercetin is a flavonoid compound that can be extracted from the Chinese herb *Sophora subprostrata* [144]. The *in vivo* experiment results of Liu Y *et al*. show that quercetin induces ER stress in OC cells through the p-STAT3/Bcl-2 pathway while promoting mitochondrion-mediated apoptosis and protective autophagy [145]. Its combination with 3-MA has been proven *in vitro* experiments to enhance the anti-cancer effect. Raddeanin A is a triterpene saponin compound in Raddeana [146]. On one hand, it inhibits the proliferation of OC Skov3 cells by regulating the relevant signaling pathway, causing the cell cycle to arrest at the G2 phase [147]. On the other hand, it inhibits autophagy by combining with antioxidants NAC or 3-MA, weakening its protective effect on autophagy in cancer cells, and thereby improving drug sensitivity [148] (Table **8**).

Therefore, for OC patients, the treatment plan combining autophagy inhibitors and natural active monomers, which leverage their high efficiency and low toxicity, is expected to become a potential option for the treatment of OC in the future.

### 4.3 Quelling Cancer's Shield: How Herbal Monomers Disarm Protective Autophagy in Ovarian Tumors

Currently, the treatment of OC is facing significant challenges. To a large extent, this is because, as the duration of drug use increases, cancer cells gradually develop drug resistance, thereby greatly reducing the clinical benefits for OC patients and putting them in an awkward situation [149]. Studies have shown that drug-targeted therapy, radiotherapy, and chemotherapy may activate autophagy. To a certain extent, this activation can become an important mechanism for the formation of drug resistance by reducing immune activity or enhancing the survival ability of cancer cells in a hypoxic environment [150]. However, it is encouraging that studies have shown that natural active components of TCM, including epigallocatechin gallate and costunolide, can inhibit the late protective autophagy of tumor cells and promote cancer cell apoptosis. More notably, some drugs can change the drug sensitivity by reducing the autophagic activity of drug-resistant strains. However, the regulatory effects of these naturally active compounds on the drug resistance of OC vary depending on the conditions, similar to the diverse roles of autophagy in OC. Perhaps by inhibiting its protective autophagy, the sensitivity of cancer cells to drugs can be increased, and drug resistance can be reduced. Overall, this brings new opportunities and challenges for the treatment of OC.

Epigallocatechin gallate is a catechin monomer isolated from tea leaves [151]. As its concentration increases, the expression level of Beclin-1 protein gradually decreases, indicating that it can exert a proliferation inhibitory effect on SKOV3 cells by inhibiting protective autophagy. Its combination with chemotherapy drugs such as cisplatin can enhance cytotoxicity and reduce the drug resistance of cancer cells, thereby significantly improving the therapeutic effect [152]. The above-mentioned drugs inhibit the proliferation of OC cells and promote the apoptosis of cancer cells by inhibiting protective autophagy. More interestingly, based on their anti-tumor effects, some other TCM monomers have played a significant role in avoiding drug resistance and improving drug sensitivity in patients. Costunolide and isoalantolactone are sesquiterpene lactones that naturally exist in *Aucklandia lappa Decne* [153]. Costunolide may inhibit the protective autophagy of OC cells by inhibiting the AMPK/mTOR signaling pathway. Isoalantolactone can significantly reduce the viability and cloning ability of OC cells and arrest the cell cycle at the G2/M phase. Its combination with cisplatin can significantly downregulate the protein expression of Mcl-1, inhibit the cell protective autophagy induced by cisplatin, and reduce the drug resistance of cancer cells. Similarly, the combination of costunolide and cisplatin can also enhance the efficacy of cisplatin *in vivo* on OC xenograft tumors and increase the sensitivity of cancer cells [154, 155]. Magnoflorine is an alkaloid extracted from Magnolia [156]. It can not only promote the apoptosis of cisplatin-resistant OC cells but also inhibit the protective autophagy induced by cisplatin, thereby enhancing the sensitivity of OC cells to cisplatin [157]. Shikonin is a naphthoquinone compound mainly extracted from *Arnebiae Radix* [158]. It possesses multiple biological activities, including anti-inflammatory, anti-viral, and anti-tumor effects. Shikonin enhances the sensitivity of SKOV3 cells to cisplatin and inhibits cell proliferation and invasion. The mechanism might be the inhibition of autophagy mediated by the Keap1/Nrf2 signaling pathway [159]. Baohuoside I is another flavonoid natural compound derived from *Epimedium* [160]. Contrary to Icariside II, *in vitro* and *in vivo* experiments have confirmed that it can downregulate the HIF-1α/ATG5 axis when combined with DDP. This combination inhibits autophagy in A2780/DDP cells, making the cells sensitive to DDP, reducing cell survival and proliferation, and inducing apoptosis [161]. Naringin can be extracted from *Rhizoma Drynariae* and *Fructus Aurantii* [162]. It not only inhibits the autophagy of SKOV3/DDP cells by regulating the PI3K/AKT/mTOR signaling pathway but also promotes their apoptosis by targeting ER stress. Naringin can reverse the drug resistance in SKOV3/DDP cells through these two mechanisms [163] (Table **9**).

In conclusion, the combined pretreatment of the aforementioned natural active components of TCM and related drugs offers a new approach to treating OC, aiming to shorten the treatment course and reduce the dose. TCM monomers and their derivatives have crucial value in overcoming the clinical drug resistance of OC by increasing or decreasing its autophagic activity and promoting or inhibiting the chemotherapy sensitization of drug-resistant strains.

## 5 DISCUSSION

TCM compounds have been widely used in the adjuvant treatment of various cancers. Autophagy is a fundamental mechanism by which cells respond to internal and external environmental stress. It plays a common role in maintaining cellular homeostasis across different types of cancer. For instance, curcumin exerts antitumor effects by regulating autophagy through the AMPK/mTOR signaling pathway in colorectal [164] and laryngeal [165] cancers. In addition, ginsenoside Rg3 has demonstrated regulatory effects on autophagy in carcinoma of the colon [166] and lung cancer [167]. The common core mechanism lies in the fact that ginsenoside Rg3 regulates Beclin-1 and P62 through multiple targets and pathways. Of course, the regulation of autophagy by individual Chinese herbal medicines varies depending on the type of cancer. For example, berberine primarily suppresses autophagy in colorectal cancer by inhibiting ATG5 expression [168]. In liver cancer, however, berberine suppresses autophagy by increasing P62 levels [169]. While the end result is the suppression of autophagy, the molecular targets involved differ due to the different expression profiles of autophagy-related genes in colorectal and liver cancers. Nevertheless, the core effect of suppressing autophagy remains unchanged. These results highlight the potential of using single herbal compounds to treat cancer through autophagy.

This text comprehensively analyzes the role and mechanism of TCM components in regulating the autophagic activity of OC. Autophagy is a type II programmed cell death that plays a crucial role in the intracellular material circulation and reuse process and is closely related to multiple aspects of tumor growth, apoptosis, invasion, metastasis, and drug resistance. However, in OC, when the autophagy of different TCM monomers is overly activated, it can cause autophagic cell death through multiple pathways, thereby promoting cell apoptosis and inhibiting the growth, progression, and metastasis of tumor cells. Pseudolaric acid B, cepharanthine, tetrandrine, matrine, harmine, lycorine, ellagic acid, astragaloside II, luteolin, and juglone can induce autophagy in OC cells by inhibiting the PI3K/AKT/mTOR signaling pathway, thereby accelerating cell death [22, 46, 54, 56, 58, 59, 68, 80, 92, 114, 119].

The various signaling pathways do not exist in isolation. The PI3K/AKT/mTOR pathway, for example, can regulate the nuclear localization of FOXO3 through phosphorylation. This forms an antagonistic relationship with the AMPK pathway: AMPK activation inhibits mTOR, while AKT has the opposite effect [170]. Furthermore, JAK2/STAT3 activation can increase HIF-1α levels, boost ATG gene expression, and work together with the MAPK/JNK pathway to promote the synthesis of autophagy-related proteins [171]. For instance, triptolide hinders the interaction between JAK2/STAT3 and HIF-1α, leading to a decrease in excessive autophagy activation [51]. In addition, the Keap1-Nrf2 pathway is considered to be the principal protective response to oxidative and electrophilic stresses [172]. These signaling pathways play a significant role in cancer. Altering one pathway can trigger a “domino effect,” resulting in various forms of cell death, including apoptosis, ferroptosis, and pyroptosis. Unfortunately, existing research has mostly focused on individual pathways, so the cross-regulatory signaling pathway network remains unclear. The following analysis focuses on how Chinese herbal medicine monomers can regulate autophagy in ovarian cancer through different signaling pathways.

Differently, both cepharanthine and tetrandrine can act synergistically with 3-MA [54, 56]. Interestingly, matrine can not only induce early lethal autophagy but also inhibit late protective autophagy [58, 59], and Polyphyllin I has the same effect [88, 89]. Moreover, luteolin can inhibit the expression of the key enzyme PARP1 of cisplatin-induced autophagy, suppress cisplatin-induced protective autophagy, and enhance the efficacy of cisplatin [114, 115].

Some monomers can promote autophagy by regulating the mTOR pathway. For instance, Eclalbasaponin II inhibits the JNK, p38, and mTOR signaling pathways [84]; Macranthoside B controls the gene expression upstream of cell apoptosis through the ROS/AMPK/mTOR pathway [86]; Icariside II can regulate the AKT/AMPK/mTOR signaling pathway and the miR-144-3p/IGF2R axis [94]; Cardamomin promotes the formation and degradation of autophagosomes through the AMPK/S6K1/mTOR-mediated signaling pathway after inhibiting glycolysis in SKOV3 cells [96, 97]. Protocatechuic acid can inhibit the phosphorylation of p70s6k by inhibiting mTORC1, thereby affecting the formation of the double membrane [48].

Unsurprisingly, based on the multi-pathway characteristics of OC treatment, several other pathways can also play a role. Triptolide enhances the autophagic flux mediated by Beclin-1 by inhibiting the JAK2/STAT3 pathway, thereby increasing the drug sensitivity of OC chemotherapy-resistant cells [50]. Salidroside may regulate KRT17 expression and be combined with paclitaxel to improve the sensitivity of SKOV3 cells to paclitaxel [33]. Neferine acts through the p38 MAPK / JNK pathway [62], and dictamnine induces cell autophagy by regulating KEAP1 expression to promote apoptosis [64]. Nobiletin can induce lethal autophagy by regulating ROS [100, 101]; Hyperoside induces autophagic death dependent on the PGRMC1/Akt pathway and partially dependent on autophagy-induced apoptosis [32]. 19-Hydroxybufotalin can induce mitochondrial autophagy [66], while berberine may promote ER stress in SKOV3 cells through autophagy, triggering a series of pro-death mechanisms [70]. Additionally, Kaempferol causes Ca^2+^ accumulation, thereby activating endoplasmic reticulum stress-mediated cytotoxic autophagy [73]. Baicalin can cause lethal autophagy and apoptosis in non-drug-resistant OC cells. When combined with cisplatin, it induces Beclin-1-independent autophagy, leading to cell death [154]. Damnacanthal inhibits tumor development by inducing autophagy through the inhibition of the ERK/mTOR signaling pathway [121]. Muscone inhibits SHH signaling to induce autophagy and promote cancer cell death [124]. Other monomers can induce autophagy by affecting related autophagy proteins, thereby exerting their anti-tumor effects.

Some TCM components may induce protective autophagy in OC to a certain extent. This autophagy helps cancer cells resist external pressure, delay apoptosis, and enhance their drug resistance. Just like the above-mentioned monomers that promote lethal autophagy, some monomers also act through the mTOR signaling pathway. For example, curcumin, paeonol, and nitidine chloride can induce protective autophagy by inhibiting the AKT/mTOR pathway [131-133, 135]. Daphnetin triggers ROS-induced cell death and induces protective autophagy by inhibiting the AMPK/Akt/mTOR pathway [141]. Chrysin increases autophagic flux [137], and Vernolepin induces protective autophagy through the p21 protein [139]. Baicalein treatment increases the phosphorylation of ERK and AKT, thereby inducing protective autophagy [143]. Quercetin induces ER stress in OC cells through the p-STAT3/Bcl-2 pathway, while promoting mitochondrial-mediated apoptosis and protective autophagy [145]. Raddeanin A can inhibit autophagy by combining with the antioxidant NAC or 3-MA, weakening its protective effect on autophagy in OC cells, and thereby increasing the sensitivity of OC to drugs [147, 148]. Compared with the above-mentioned lethal autophagy, this autophagy may cause drug resistance in cancer cells to a certain extent, but we do not need to be overly worried. The protective autophagy caused by the above monomers can be weakened or eliminated by combining with autophagy inhibitors, thereby enhancing the anti-tumor effect. Even in this way, it has opened a new door for improving the sensitivity of chemotherapy drugs.

Some monomers can inhibit protective autophagy, promote cancer cell apoptosis, improve drug sensitivity, and reduce drug resistance while exerting their anti-tumor effects. Naringin inhibits the autophagy of SKOV3/DDP cells by regulating the PI3K/AKT/mTOR signaling pathway [163]. Costunolide may inhibit the protective autophagy of OC cells by inhibiting the AMPK/mTOR signaling pathway [155]. Costunolide may inhibit the protective autophagy of OC cells by inhibiting the AMPK/mTOR signaling pathway [154]. Shikonin may be able to inhibit autophagy mediated by the Keap1/Nrf2 signaling pathway [159]. In addition, Baohuoside I, combined with DDP, inhibits autophagy in A2780/DDP cells by downregulating the HIF-1α/ATG5 axis, which makes the cells sensitive to DDP, reduces cell survival and proliferation, and induces apoptosis [161].

When discussing the role of TCM monomers in the treatment of OC, we must first recognize that autophagy has a dual role in cell survival and death. Autophagy is not only a protective mechanism that helps cells survive under stress, but it may also have adverse consequences; it can also act as a cell death pathway that promotes the clearance of tumor cells. Autophagy exhibits distinct stage-specificity in tumor progression, with different roles in the early and late stages. In the early stages of tumor development, autophagy is usually suppressed. This suppression facilitates tumor cell proliferation and survival because the inhibition of autophagy reduces the metabolic burden on cells, promoting rapid cell division [173]. Additionally, suppressing autophagy helps tumor cells evade immune surveillance. Studies have shown that autophagy plays a role in multiple steps of the anti-tumor immune response in immune cells, including antigen presentation and regulation of inflammatory responses [174]. Therefore, inhibiting autophagy can weaken the immune system's ability to recognize and eliminate tumor cells. However, in the late stages of tumor development, autophagy may be activated. This activation helps tumor cells adapt to hypoxic and nutrient-deprived microenvironments, thereby enhancing their survival capacity. For instance, autophagy provides energy and nutrients by degrading damaged organelles and proteins, enabling tumor cells to survive and metastasize in harsh environments [175]. TCM monomers also have a dual nature in regulating autophagy, which requires us to finely regulate the treatment strategy and clarify the effective drug concentration to maximize the therapeutic effect and reduce side effects. To find the best autophagy regulation balance point, it is necessary to combine existing studies and refer to possible future clinical data to determine the median effective dose and maximum drug concentration of TCM monomers. In addition, combining with known autophagy inhibitors can enhance the anti-tumor effect, reverse protective autophagy, and improve the sensitivity of chemotherapy drugs. At the same time, exploring the synergistic effects of different TCM monomers may lead to the discovery of new treatment combinations to achieve more targeted autophagy regulation.

Compared with a large number of *in vitro* experiments, there are fewer studies on the mechanism by which TCM components regulate autophagy in OC *in vivo*. Unfortunately, no relevant data have been found in this article regarding clinical experiments, which may be due to the narrow application scope of molecular TCM in such studies. However, we hope that the discussion in this article can attract more attention to this unknown field, make full use of the natural medicine treasury, break through the limitations of existing drugs, and expand the treatment strategies for OC.

We look forward to the breakthroughs that the multi-center, randomized controlled clinical trials designed in the future can achieve in the following aspects: 1) to cooperate with traditional chemotherapy drugs to improve chemotherapy sensitivity and exert their synergistic effects; 2) through clinical comparisons, to screen out effective TCM monomers that can delay the progression of the disease and prolong the survival of patients; 3) to use TCM monomer preparations to observe whether they can reduce the toxic and side effects of chemotherapy, and realize the beautiful hope of enhancing patients' confidence in survival and improving their quality of life. At the same time, stratification can be carried out according to the specific situation of patients (such as genotype, disease stage, *etc*.) to determine which patient groups are most likely to benefit from TCM monomer preparations. In clinical trial research, tumor tissue samples and ascites samples can be collected in subsequent experiments, while peripheral blood samples can be used in clinical trials to detect autophagy-related molecular markers and assess autophagic activity [176]. However, the specific detection method is not yet clear, but we believe that with the deepening of our understanding of the autophagy pathway, it may be possible to develop radiological techniques (such as positron emission tomography) that can detect autophagy or have clinical value [177].

Through the above elaboration, it is not difficult to see that the role of autophagy in tumors is complex. It not only has the potential to inhibit tumors but also can play a promoting role in tumor progression. Therefore, more in-depth studies are needed to clarify the specific role of autophagy in different stages. Future studies should further explain the molecular mechanisms by which TCM components regulate autophagy, efficiently separate the specific effective components, accurately determine their effective therapeutic concentrations, and apply them to clinical studies of OC to verify their effectiveness and safety, thereby providing new methods for delaying the progression of OC. In addition, the combined pretreatment of TCM components and chemotherapy drugs offers a more effective approach to treating OC and is expected to become a potential choice in the future.

TCM monomers may attract more researchers to study their regulation of autophagy in OC due to their multi-target effects, high safety, low toxicity and side effects, ability to reduce patients' economic burden, and potential for reversing drug resistance. They can control the entire process of autophagy in OC cells by influencing multiple signaling pathways and biological processes, achieving a comprehensive inhibition of tumor cells. However, these need to be confirmed through strict scientific verification and are expected to be fully applied in clinical practice.

## CONCLUSION

In recent years, the role of autophagy in OC has garnered increasing attention as medical research on OC treatment has expanded. This review indicates that single Chinese herbal medicine components can influence the occurrence and development of OC by regulating the autophagy process, exhibiting bidirectional effects. For example, components such as ginsenoside Rg3 and curcumin can bidirectionally regulate autophagy levels through signaling pathways like PI3K/AKT/mTOR and AMPK. They can induce excessive autophagy to trigger cell death and inhibit protective autophagy to enhance chemotherapy sensitivity. However, existing studies are primarily based on *in vitro* cell experiments (*e.g.*, SKOV3, A2780 cell lines), and such models lack the complex tumor microenvironment (*e.g.*, cytokines in ascites fluid, fibroblast infiltration), making it difficult to simulate the clinical characteristics of OC peritoneal metastasis. Animal models primarily use immunodeficient mice for xenografting, which can reflect tumor growth trends but cannot fully replicate the dynamic regulatory relationship between the human immune system and autophagy. This limitation may lead to discrepancies between the dose-response effects of herbal medicine components and clinical reality. While these studies provide potential directions for the development of natural drugs targeting autophagy, there is still a lack of sufficient clinical research to support their precise mechanisms of action and efficacy in humans. We believe that through carefully designed experimental protocols and clinical trials, we may uncover the specific mechanisms of action and potential synergistic effects of TCM single components in OC treatment. Given their unique properties and multifaceted mechanisms of action, they represent promising candidate drugs worthy of further investigation. In the future, they may be seamlessly integrated with modern formulation technologies, potentially opening up broader therapeutic prospects in the field of OC and improving patient outcomes and quality of life.

## AUTHORS’ CONTRIBUTIONS

The authors confirm their contribution to this paper as follows: M-Y G is responsible for writing the first draft of the paper and making several revisions and refinements with the assistance of other authors. Y-F Z, YY, D-N D, and YW were responsible for guiding the revision of the paper and put forward many valuable suggestions. F-J H oversaw the writing and final review of the paper and provided funding support. All authors contributed to the article and agreed to the final version of the paper.

## Figures and Tables

**Table 1:** Mechanism of natural terpenoids in the autophagy of OC.

**Drug Name**	**Source**	**Concentration/Time**	**Cell Lines and Animal Models**	**Pharmacological Mechanisms**	**Effect Results**	**Verify**	**References**
Pseudolaric acid B	golden larch bark	*in vitro* (0, 2, 4, 8μmol/L)/ (0, 6, 12, 24h)	HO8910	LC3-II/LC3-I, LC3-I, **Beclin-1**↑ p-AKT, p-mTOR↓	Concentration-time dependent Autophagy↑ HO8910 cell survival rate ↓	3-MA, RAPA	[46]
Poria acid A	Poria cocos	*in vitro* (0, 30, 50, 80μg/mL)/24h	SKOV3	LC3-II/LC3-I↑ **p-mTOR**, p-p70s6k ↓	Concentration-time dependent Autophagy, Apoptosis↑ Proliferation, Migration, Invasion ↓	MHY1485	[48]
Triptolide	Tripterygium wilfordii	*in vitro* (0, 25, 50, 100nM)/ (12, 24h) *in vivo* (Triptolide intraperitoneal injection: 0.15mg/kg/d, once a day, 10 times in total)	SKOV3/DDP, Nude mouse SKOV3/DDP ovarian cancer subcutaneous xenograft model	LC3-II/LC3-I, Beclin-1, Beclin-1 mediated autophagic flux ↑ p-JAK2/JAK2, p-STAT3/STAT3, Beclin-1/Mcl-1 compound, P62↓	*in vitro*: Concentration-time dependent Autophagy, Survival rate of SKOV3 / DDP cells ↑ Proliferation ↓ *in vivo*: The Triptolide + DDP group showed the most significant reduction in tumor volume and weight. cleaved caspase-3, LC3, Sensitivity to cisplatin↑ Ki67, P62 ↓	3-MA, CQ, AG490, IL-6	[50, 51]
Salidroside	Rhodiola	*in vitro* SAL(0, 100, 200μmol/L)± PTX (0, 10, 20nmol/L)/24h	SKOV3	Autophagosome, LC3-II/LC3-I, Beclin-1↑ KRT17, P62↓	Concentration-time dependent Autophagy, Cytotoxicity, Apoptosis rate, sensitivity of paclitaxel to SKOV3 cells ↑ Survival rate and proliferation of SKOV3 cells ↓	-	[33]

**Notes:** ↑ arrows for increase, growth, elevation, promotion; ↓ arrow represents decrease, diminish, lower, inhibit. All female mice were used in the *in vivo* experiments.

**Table 2:** Mechanism of alkaloids in the autophagy of OC.

**Drug Name**	**Source**	**Concentration/Time**	**Cell Lines and Animal Models**	**Pharmacological Mechanisms**	**Effect Results**	**Verify**	**References**
Cepharanthine	Stephania japonica	*in vitro* (0, 2, 4, 8, 16, 32μmol/L) /24h	SKOV3	LC3-II ↑ p-AKT, p-mTOR↓	Optimal concentration:8, 16μmol/L autolysosome↑ The viability of SKOV3 cells (with a better-combined effect when used with 3-MA) ↓	3-MA	[54]
Tetrandrine	Stephania tetrandra	*in vitro* (0, 2, 4, 8, 16 µmol/L) / 24 h	SKOV3	LC3-II, P62↑ p-mTOR, p-AKT↓	Concentration-dependent At 4 and 8 µmol/L, autolysosomes↑ The viability of SKOV3 cells (with a better combined effect when used with 3-MA) ↓	3-MA	[56, 57]
Matrine	Sophora flavescens	*in vitro* (2.0mg/mL)/24h	A2780, SKOV3	LC3-II ↑ p-AKT, p-mTOR, SQSTM1/P62↓	Concentration-dependent Autophagosome, Autolysosome, Early autophagy, The sensitivity of cells to chemotherapeutic drugs↑ Late autophagy↓	-	[59]
Harmine	Peganum harmala	*in vitro* (0, 20, 30μM) /48h *in vivo* Intraperitoneal injection (60 mg/kg/day) for 24 days	SKOV3 Ten Female SCID mice were injected with 5 × 10^6^ SKOV3 cells subcutaneously into the right flanks.	LC3-II, LC3-II/LC3-I, ATG5, Beclin-1, FOXO3↑ P62, p-mTOR, p-PI3K, p-AKT↓	*in vitro*: The number of autophagic vesicles, Autophagosome↑ *in vivo*: Significant reduction in tumor volume Number of apoptotic cells, ATG5↑ cell density, GPX4, NLRP3, Ki-67↓	3-MA, BafA1	[22]
Neferine	Lotus Plumule	*in vitro* (5, 10μM)/24h	A2780	LC3-II, ATG7, p-P38, p-JNK↑ p70S6K, 4EBP1↓	Concentration-dependent Apoptosis, Autophagy, Autophagosome↑ Proliferation↓	SB203580 (P38 inhibitors), SP600125 (JNK inhibitors)	[62]
Dictamnine	Dictamnus dasycarpus Turcz	*in vitro* (0, 50, 75μmol/L)/ (48, 72 h)	A2780	LC3-II/LC3-I↑ P62, KEAP1, PI3K↓	Concentration-dependent Apoptosis, Autophagy↑ Proliferation↓	-	[64]
19-Hydroxytelocinobufagin	Toad	*in vitro* (0, 25, 50, 100, 200, 300, 400nM)/ (24, 48 h)	A2780, SKOV3	Beclin-1, ATG5, LC3-II, Parkin, ROS ↑ P62↓	concentration-time dependence Cell cycle arrest at S phase and G2/M phase, Apoptosis, Mitochondrial autophagy↑ Survival rate, Proliferation↓	-	[66]
lycorine	Lycoris genus	*in vitro* 25ug/ml /72h	SKOV3	AKT, PI3K, ATG5, P53, LC3-II↑ Bcl-2, P-AKT, P62↓	concentration-time dependence G0/G1 phase, Apoptosis, Autophagy↑ Invasion, Migration↓	-	[68]
Berberine	Coptis chinensis	*in vitro* 200μmol/L/24 h	SKOV3	CHOP, P62, Beclin-1, LC3-II, ATG5↑ GRP78, PERK, ATF6, p-ATG↓	concentration-time dependence Cytotoxic autophagy↑ Cell viability↓	-	[70]

**Notes:** ↑ arrows for increase, growth, elevation, promotion; ↓ arrow represents decrease, diminish, lower, inhibit. All female mice were used in the *in vivo* experiments.

**Table 3:** Mechanism of natural dietary polyphenols in the autophagy of OC.

**Drug Name**	**Source**	**Concentration/Time**	**Cell Lines and Animal Models**	**Pharmacological Mechanisms**	**Effect Results**	**Verify**	**References**
Kaempferol	*Kaempferia galanga, Scutellaria barbata*	*in vitro* 40µmol/L /24h	A2780	Ca^2+^, LC3-II, Beclin-1, ATG5↑	Apoptosis↑ Survival rate, Proliferation↓	-	[73]
Resveratrol	Veratrum	*in vitro* 25µmol/L /24h	SKOV3, OVCAR3	LC3-II, Beclin-1, ATG5, P62, ROS↑	Concentration-dependent Apoptosis, Autophagy↑ Proliferation, Autophagic flux↓	3-MA, CQ	[75, 76]
Protocatechuic acid	*Suberect spatholobus*	*in vitro* (20, 25μM)/12h	OVCAR3	LC3-II↑	Apoptosis, Autophagy↑	3-MA、CQ	[78]
Ellagic acid	Euphorbia, gallnuts	*in vitro* 36.6µmol/L /24h	SKOV3	Beclin-1, ATG5, LC3-II, p-AMPK, p-mTOR↑ P62, p-AKT, p-S6K1 ↓	Concentration-dependent Apoptosis, Cytotoxic autophagy↑ Survival rate, Proliferation, Invasion, Migration↓	CQ	[80]

**Notes: **↑ arrows for increase, growth, elevation, promotion; ↓ arrow represents decrease, diminish, lower, inhibit. All female mice were used in the *in vivo* experiments.

**Table 4:** Mechanism of saponins in the autophagy of OC.

**Drug Name**	**Source**	**Concentration/Time**	**Cell Lines and Animal Models**	**Pharmacological Mechanisms**	**Effect Results**	**Verify**	**References**
Ginsenoside 20 (S)-Rg3	ginseng	*in vitro* (10, 20, 40, 80μg/ml)/ (24, 48h)	SKOV3	ATG5, ATG7, LC3- II/ LC3-I ↑ p-AKT, AKT, mTOR↓	Concentration-dependent Autophagy-lysosome, Autophagy↑ Proliferation↓	CQ	[82]
Eclalbasaponin II	Eclipta prostrata	*in vitro* (10, 12.5, 20, 25, 30μM)/48h	SKOV3, A2780	LC3-II, Beclin-1, p-P38, p-JNK↑ P62, p-mTOR↓	Concentration-dependent Cytotoxic Autophagy, Apoptosis↑ Proliferation↓	BafA1	[84]
Macranthoside B	Lonicera macranthoides	*in vitro* (10, 20 mM)/48h	A2780	ROS, LC3-II, p-AMPK↑ p-mTOR, p-p70S6↓	concentration-time dependence Cytotoxic autophagy, Apoptosis↑ Proliferation↓	3-MA	[86]
Polyphyllin I	Paris polyphylla	*in vitro*(0, 0.1, 0.5, 1, 2, 5, 10μM)/24h *in vivo*((10, 20, 30 mg/kg) Subcutaneous injection once every 2 days, repeated 7 times)	SKOV3 Female C57 mice were inoculated subcutaneously in the right axilla with 0.1 mL of ID8 cells (cell count: 3.25 × 10⁶ cells)	LC3-II↑ P62↓	Concentration-dependent *in vitro*: Apoptosis, Autophagy↑ Proliferation, Tumor volume, Late-stage autophagy↓ *in vivo*: Mouse weight, Mouse activity, Coat luster, Tumor volume↓	-	[88, 89]
Saikosaponins	Bupleurum	*in vitro* (0, 10, 20μmol/L)/ (24, 48, 72 h)	SKOV3	LC3-II/LC3-I, Beclin-1↑	Concentration-dependent Apoptosis, Autophagy↑ Proliferation↓	-	[91]
Astragaloside II	Astragalus	*in vitro* (160, 320, 640μM) /48h *in vivo* (Mice were administered intraperitoneal injections of 5 mg/kg or 10 mg/kg daily, or combined intraperitoneal injections of DDP 1 mg/kg every other day)	SKOV3/DDP, A2780/DDP Inoculate 5×10⁶ SKOV3/DDP and A2780/DDP cells into the dorsal subcutaneous tissue of female BALB/c-nu mice to achieve a tumor volume of 100–150 mm³.	LC3-II↑ P62, p-mTOR, p-AKT↓	Concentration-dependent *in vitro:* Apoptosis, Autophagy, and the sensitivity of DDP ↑ *in vivo:* SKOV3/DDP and A2780/DDP tumor volume, PCNA expression levels↓	-	[92]

**Notes:** ↑ arrows for increase, growth, elevation, promotion; ↓ arrow represents decrease, diminish, lower, inhibit. All female mice were used in the *in vivo* experiments.

**Table 5:** Mechanism of flavonoids in the autophagy of OC.

**Drug Name**	**Source**	**Concentration/Time**	**Cell Lines and Animal Models**	**Pharmacological Mechanisms**	**Effect Results**	**Verify**	**References**
Icariside II	Epimedium	*in vitro* (0, 20, 40, 60 μM)/48h *in vivo* (administered 25 mg/kg icastatin II daily* via *a gastric tube.)	SKOV3, A2780 BALB/c nude mice (6 weeks old) were injected subcutaneously with 100 μL of A2780 cell suspension (5 × 10^6^ cells/mL)	miR-144-3p, Beclin-1, ATG5 ↑ P62, p-mTOR, p-AKT, IGF2R↓	*In vitro*: Concentration-dependent Apoptosis, Autophagy↑ Proliferation, Invasion, Migration↓ *in vivo*: Tumor weight↓	-	[94]
Cardamomin	Alpinia katsumadai	*in vitro* (5, 10, 20μM)/48h	SKOV3	LC3-II, p-AMPK↑ p-AKT, p-mTOR, p-S6K1 , HK2, LDH, ATP↓	Concentration-dependent Autophagy↑ Glycolysis, Proliferation↓	-	[96]
Diosmetin	Bergamot, Chrysanthemum	*in vitro* (0, 10, 20, 40μmol/L)/48h	A2780, SKOV3	LC3-II↑ P62↓	Concentration-dependent Apoptosis, Autophagy↑ Proliferation, Invasion, Migration↓	-	[99]
Nobiletin	Fructus Aurantii	*in vitro* (0, 10, 30, 50μM)/24h	A2780, OVCAR, SKOV3/TAX	ROS, LC3-II, P62, p-AKT, p-mTOR, p-p70S6K↑	Concentration-dependent Autophagosome, Mitophagosome, Apoptosis, Autophagy, Pyroptosis↑ Proliferation, Autophagy, lysosomes, Autophagic flux↓	3-MA, NAC	[100, 101]
Garcinone E	Garcinia oblongifolia	*in vitro* 5μM/24h	A2780	LC3-II, P62↑ Rab7, Beclin-1 ↓	Concentration-time dependence Apoptosis, Autophagy, autophagosomes↑ Autophagy lysosomes, Autophagic flux↓	-	[103]
Trichosanthin	Radix Trichosanthis	*in vitro* (3.3, 6.7, 10μM)/48h	OVCAR3	ATG5, LC3-II↑	Concentration-time dependence Apoptosis, Autophagy↑ Proliferation↓	3-MA	[105]
Isoliquiritigenin	Glycyrrhiza uralensis	*in vitro* (10, 25, 50μM)/48h	OVCAR5	LC3-II, PARP, Beclin-1↑ P62↓	Optimal concentration: 25μM. Apoptosis, Autophagy↑ , Proliferation↓	3-MA	[107]
Myricetin	Myrica rubra	*in vitro* (0, 20, 40, 60g/L)/12h	SKOV3	LC3-II/LC3-I↑	Concentration-dependent Mitochondrial fission, Apoptosis, Autophagy↑ Mitochondrial membrane potential↓	3-MA	[109]
Hyperoside	Hypericaceae	*in vitro* (0, 50, 100μM)/ (24, 48, 72h)	SKOV3, HO-8910	LC3-II, PGRMC-1↑ p-AKT↓	Concentration-time dependence Apoptosis, Autophagy, Sensitivity of cancer cells to DDP↑ Proliferation↓	3-MA	[32]
Baicalin	Scutellaria baicalensis	*in vitro* (200-400μg/mL)/24h	A2780 (Cancer Stem Cell , CSC)	ATG5, ATG12↑	CSC apoptosis and autophagic death were caused by a single use↑	-	[112]
Luteolin	Bamboo leaves	*in vitro* (0, 10, 20μmol/L)/48h	A2780	LC3-II/LC3-I↑ P62, p-AKT, p-mTOR, Bcl-2↓	Concentration-dependent Apoptosis, Autophagy↑ Cell viability, Invasion, Migration, Proliferation↓	-	[114, 115]

**Notes:** ↑ arrows for increase, growth, elevation, promotion; ↓ arrow represents decrease, diminish, lower, inhibit. All female mice were used in the *in vivo* experiments.

**Table 6:** Mechanism of quinones in the autophagy of OC.

**Drug Name**	**Source**	**Concentration/Time**	**Cell Lines and Animal Models**	**Pharmacological Mechanisms**	**Effect Results**	**Verify**	**References**
Tanshinone IIA	*Salvia miltiorrhiza*	*in vitro* (0.4, 0.6, 0.8, 1.2, 1.6, 1.8, 3.2mg /L)/24h	A2780	Beclin-1, LC3-II/LC3-I↑	Concentration-dependent Apoptosis, Autophagy↑ Invasion, Migration, Proliferation↓	-	[117]
Juglone	*Juglans regia*	*in vitro* (25, 50, 100μmol/L)/48h	SKOV3	Beclin-1, ATG7, LC3-II↑ p-AKT, p-mTOR, P62↓	Concentration- dependent Autophagy↑ Proliferation↓	-	[119]
Damnacanthal	Rubiaceae	*in vitro* (0, 5, 10, 20μM) / (0, 12, 24, 48h) *in vivo*: Intraperitoneal injection: Damnacanthal (1, 5mg/kg), Cisplatin (3 mg/kg), 3 times a week for 2 weeks	SKOV3, A2780 Inject 2 × 10⁶ SKVO3 cells subcutaneously into the right axilla of female BALB/c nude mice.	LC3-II↑ P62, p-AKT, p-mTOR↓	*In vitro*: Concentration-dependent Apoptosis, Autophagy↑ Invasion, Migration↓ *in vivo*: Dose-dependent manner Tumor cells are loosely arranged and irregular in shape. Apoptosis↑ The volume of tumor Nodules, Tumor growth↓	3-MA	[121]

**Notes: **↑ arrows for increase, growth, elevation, promotion; ↓ arrow represents decrease, diminish, lower, inhibit. All female mice were used in the *in vivo* experiments.

**Table 7:** Mechanism of other compounds in the autophagy of ovarian cancer.

**Drug Name**	**Source**	**Concentration/Time**	**Cell Lines and Animal Models**	**Pharmacological ** **Mechanisms**	**Effect Results**	**Verify**	**References**
Muscone	Musk	*in vitro* (16μmol/L/48h) *in vivo*: Subcutaneous injection: 16 mg/kg muscone at the tumor site, once daily for a total of 28 days.	SKOV3 1 × 10⁷/mL SKOV3 cells were inoculated subcutaneously into the right axilla of BALB/c mice, 0.1mL/mouse.	LC3-II/LC3-I, Beclin-1↑ SHH mRNA ↓	*in vitro*: Apoptosis, Autophagy↑, Proliferation, Invasion, Migration↓ *in vivo*: Tumor volume and mass↓	CQ	[124]
Pinoresinol	*Forsythia suspensa, Eucommia ulmoides*	*in vitro* (0, 10, 20, 40µM)/24h *in vivo*: Intraperitoneal injection: Pinoresinol 40 mg/kg, 3 times/week, for a total of 6 weeks.	SKOV3 5×10^6^ SKOV-3 cells were injected subcutaneously into the left abdomen of 4-week-old mice.	Beclin-1, LC3-II↑ P62, MMP, p-MEK, p-ERK↓	*in vitro*: Concentration-dependent Autophagy↑ Proliferation, Invasion, Migration↓ *in vivo*: Tumor volume and mass↓	-	[126]
Osthole	*Cnidium monnieri, Angelica sinensis*	*in vitro* (0, 20, 40, 80 μM)/24h	A2780, OVCAR3	LC3-II, ROS↑ MMP↓	Concentration-dependent Apoptosis, Autophagy↑ Proliferation, Invasion, Migration↓	-	[128]

**Notes:** ↑ arrows for increase, growth, elevation, promotion; ↓ arrow represents decrease, diminish, lower, inhibit. All female mice were used in the *in vivo* experiments.

**Table 8:** Mechanism of TCM monomer promoting protective autophagy in OC.

**Drug Name**	**Source**	**Concentration/ Time**	**Cell Lines and Animal Models**	**Pharmacological Mechanisms**	**Effect Results**	**Verify**	**References**
Curcumin	Turmeric	*in vitro* SKOV3 (0, 10, 20, 40 μM) *in vitro* A2780(0, 7.5, 15, 30 μM)/ (24, 48, 72h)	A2780, SKOV3	Beclin-1, LC3-II, ATG3↑ p-PI3K, p-AKT, p-mTOR, p-p70S6K ↓	Concentration-time dependence Apoptosis (The combined use with CQ has a better effect), Protective Autophagy, sensitivity ↑ Proliferation↓	CQ	[131, 133]
Paeonol	Moutan Cortex	*in vitro* (0, 0.3, 0.6, 1.2μM)/ (24, 48h) *in vivo*: Intraperitoneal injection: Paeonol (40 mg/kg) every 2 days for a total of 6 times, then observe for 12 days.	A2780, SKOV3, A2780 cells (1 × 10^7^/0.2 mL) were injected subcutaneously into the left side of BALB/c nude mice.	LC3-II↑ P62, p-PI3K, p-AKT, p-mTOR, p-p70S6K ↓	*in vitro* Concentration-time dependence Apoptosis (with a better-combined effect when used with 3-MA and CQ), Autophagosome, Autophagic flux, Protective Autophagy, Sensitivity↑ Proliferation↓ *in vivo* Tumor volume and mass, Bcl-2, Ki-67 ↓	3-MA, CQ	[132]
Nitidine chloride	*Zanthoxylum nitidum*	*in vitro* (2, 4μM)/48 h	A2780, SKOV3	LC3-II/LC3-I↑ P62, AKT, mTOR, p-Akt, p-mTOR, p-P85S6K, p-p70S6k, p-4E-BP1↓	Concentration-time dependence Protective Autophagy, Autophagosome, Apoptosis↑ Cell viability, Migration, Proliferation↓	CQ	[135]
Chrysin	*Oroxylum indicum* (L.) Vent.	*in vitro* (0, 20, 40, 60, 80, 100μmol /L)/24h	SKOV3	LC3↑	Concentration-dependent Apoptosis (The combined use with CQ has a better effect), Protective Autophagy, Autophagosome↑ Proliferation↓	CQ	[137]
Vernolepin	*Vernonia amygdalina*	*in vitro* 30uM/24h	OVCAR3	P21, LC3↑	Concentration-dependent Apoptosis (The combined use with 3-MA has a better effect), Protective Autophagy, Autophagosome, Sensitivity↑ Proliferation↓	3-MA	[139]
Daphnetin	Daphne plants	*in vitro* (0, 10, 20, 40µg/ml)/24h *in vivo*: Intraperitoneal injection: Daphnetin (30 mg/kg), once daily for 12 days	A2780 Subcutaneously inject A2780 cells (1 × 10^7^ /mL) into the right back of BALB/c nude mice.	LC3-II, P62, p-AMPK↑ ROS, p-AKT, p-mTOR↓	*in vitro* Concentration-dependent Apoptosis (with a better-combined effect when used with 3-MA and CQ), Protective Autophagy, Sensitivity↑ Proliferation↓ *in vivo* Anti-tumor efficacy (in combination with CQ), Caspase 3↑ Tumor volume and weight, Ki-67↓	3-MA、CQ	[141]
Baicalein	*Scutellaria baicalensis*	*in vitro* (0, 12.5, 25, 50 μM)/24h	HEY cells, A2780	LC3-II, Beclin-1, GFP-LC3 puncta, p-ERK, p-AKT, ULK1↑	Concentration-dependent Apoptosis, Autophagic flux, Protective Autophagy, Sensitivity↑ Cell viability, Proliferation↓	CQ	[143]
Quercetin	*Sophora subprostrata*	*in vitro* (0, 10, 20, 40μM)/48h *in vivo*: Intraperitoneal injection: Quercetin (80 mg/kg), 2 times/week, total of 4 weeks.	CAOV3 Naked mice were intraperitoneally injected with CAOV3 (5 × 10^6^ cells/200 μL). CAOV3 cells (5 × 10^6^ cells/100 μL) were injected subcutaneously into nude mice.	LC3-II, Beclin-1, GRP78, ATG5↑ p-STAT3, Bcl-2↓	*in vitro* Concentration-dependence Endoplasmic reticulum stress, Mitochondrial apoptosis, Cytotoxicity, Protective Autophagy, Sensitivity↑ Proliferation↓ *in vivo* Quercetin combined with 3-MA treatment (Compared to using quercetin alone, it is more effective.): survival period, caspase-3↑ Abdominal tumor burden, Tumor volume↓	3-MA	[145]
Raddeanin A	Raddeanab	*in vitro* (1, 2, 4μM)/ (3, 6h)	SKOV3	ROS↑	Apoptosis, Protective Autophagy↑ Proliferation↓	3-MA, NAC	[147, 148]

**Notes:** ↑ arrows for increase, growth, elevation, promotion; ↓ arrow represents decrease, diminish, lower, inhibit. All female mice were used in the *in vivo* experiments.

**Table 9:** Mechanism of TCM monomer inhibiting protective autophagy in OC.

**Drug Name**	**Source**	**Concentration/Time**	Cell Lines and Animal Models	**Pharmacological Mechanisms**	**Effect Results**	**Verify**	**References**
Epigallocatechin gallate	Tea leaves	*in vitro* (10, 20, 40μmol/L)/24h	SKOV3	Beclin-1↓	Concentration-dependent Cytotoxicity, Sensitivity ↑ Proliferation, Protective Autophagy↓	Combined with cisplatin	[152]
Costunolide	Aucklandia lappa Decne	*in vitro* (10, 15, 20μmol/L)/24h	A2780, ES-2 cell	P62, LC3-II, p-mTOR, p-ULK1, p-S6K↑ p-AMPK, VAMP8, STX17, SNAP29↓	Concentration-dependent Apoptosis↑ Proliferation, Autophagic flux, Autolysosome, Cisplatin-induced Protective Autophagy↓	BafA1, combined with cisplatin	[155]
Isoalantolactone	Aucklandia lappa Decne	*in vitro* 12μM/24h	SKOV3	Mcl-1, LC3-Ⅱ, Beclin-1↓	Sensitivity, Cytotoxicity, Apoptosis↑ Cell viability, Proliferation, Cisplatin-induced Protective Autophagy↓	Combined with cisplatin	[154]
Magnoflorine	Magnolia	*in vitro* (0, 40μM)/24h	A2780, SKOV3, A2780/DDP, SKOV3/DDP	P62↑ LC3-II/LC3-I, Beclin-1↓	Apoptosis, Sensitivity↑ Proliferation, Cisplatin-induced Protective Autophagy↓	Combined with cisplatin.	[157]
Shikonin	Arnebiae Radix	*in vitro* 9μmol/L/48h	SKOV3	Keap1↑ Nrf2, Beclin-1, LC3-II/LC3-I, ULK1↓	Concentration-dependent Apoptosis, Sensitivity↑ Proliferation, Migration, Invasion Protective Autophagy↓	-	[159]
Baohuoside I	Epimedium	*in vitro* (0, 2.5, 5, 10, 20M)/48h *in vivo*: Intraperitoneal injection: DDP+ Baohuoside I (4 mg/kg/day DDP, 20 mg/kg/day Baohuoside I), for 3 weeks	A2780, A2780/DDP Subcutaneous injection of A2780/DDP cells (5 × 10^6^/mouse) into BALB/c nude mice.	DDP + Baohuoside I: HIF-1α, ATG5, LC3-II/LC3-I↑	*in vitro* Concentration-dependent Apoptosis, Sensitivity ↑ Cell viability, Proliferation, Protective Autophagy, Sensitivity of A2780/DDP cells to cisplatin↓ *in vivo* Tumor volume and weight, OC resistance to cisplatin, Ki67↓	-	[161]
Naringin	Rhizoma Drynariae and Fructus Aurantii	*in vitro*	SKOV3/DDP	p-PI3K, p-AKT, p-mTOR↑	Concentration-dependent Apoptosis, Sensitivity ↑ Cell viability, Proliferation, Protective Autophagy↓	BafA-1,3-MA	[163]

**Notes:** ↑ arrows for increase, growth, elevation, promotion; ↓ arrow represents decrease, diminish, lower, inhibit. All female mice were used in the *in vivo* experiments.

## LIST OF ABBREVIATIONS

3-MA: 3-Methyladenine
AKT: Protein Kinase B
AMPK: AMP-Activated Protein Kinase
ATG: Autophagy-Related Genes
BafA1: Bafilomycin A1
Bcl-2: B-cell lymphoma-2
CQ: Chloroquine
CSC: Cancer Stem Cell
ERK: Extracellular signal-regulated kinase
FOXO3: Forkhead Box O3
HIF-1α: Hypoxia-Inducible Factor 1 Alpha
IGF2R: insulin-like growth factor 2receptor
IL-6: Interleukin-6
JAK2: Janus Kinase 2
JNK: c-Jun N-terminal Kinase
Keap1: Kelch-like ECH-Associated Protein 1
KRT17: Keratin 17
LC3: Microtubule-Associated Protein 1 light chain 3
MAPK: Mitogen-Activated Protein Kinase
Mcl-1: Myeloid Cell Leukemia-1
mTOR: Mammalian Target of Rapamycin
NAC: N-Acetylcysteine
Nrf2: Nuclear Factor Erythroid 2-Related Factor 2
OC: Ovarian cancer
p70S6K: p70 Ribosomal S6 Kinase
PGRMC1: Progesterone Receptor Membrane Component 1
PI3K: Phosphatidylinositol 3-Kinase
Pink1: PTEN-Induced Kinase 1
SHH: Sonic Hedgehog
STAT3: Signal Transducer and Activator of Transcription 3
TCM: Traditional Chinese Medicine
ULK1: Unc-51 Like Autophagy Activating Kinase 1
Vps34: Vacuolar Protein Sorting 34

## References

[1] Cao W., Chen H.D., Yu Y.W., Li N., Chen W.Q. (2021). Changing profiles of cancer burden worldwide and in China: A secondary analysis of the global cancer statistics 2020.. Chin. Med. J..

[2] Webb P.M., Jordan S.J. (2024). Global epidemiology of epithelial ovarian cancer.. Nat. Rev. Clin. Oncol..

[3] Chien J., Poole E.M. (2017). Ovarian cancer prevention, screening, and early detection: Report from the 11th biennial ovarian cancer research symposium.. Int. J. Gynecol. Cancer.

[4] Webb P.M., Jordan S.J. (2017). Epidemiology of epithelial ovarian cancer.. Best Pract. Res. Clin. Obstet. Gynaecol..

[5] Havasi A., Cainap S.S., Havasi A.T., Cainap C. (2023). Ovarian cancer—insights into platinum resistance and overcoming it.. Medicina.

[6] Mizushima N., Levine B. (2020). Autophagy in human diseases.. N. Engl. J. Med..

[7] Ma X., Hu M., Wang H., Li J. (2018). Discovery of traditional Chinese medicine monomers and their synthetic intermediates, analogs or derivatives for battling P-gp-mediated multi-drug resistance.. Eur. J. Med. Chem..

[8] Levy J.M.M., Towers C.G., Thorburn A. (2017). Targeting autophagy in cancer.. Nat. Rev. Cancer.

[9] Wang S.F., Wu M.Y., Cai C.Z., Li M., Lu J.H. (2016). Autophagy modulators from traditional Chinese medicine: Mechanisms and therapeutic potentials for cancer and neurodegenerative diseases.. J. Ethnopharmacol..

[10] Thorburn A., Thamm D.H., Gustafson D.L. (2014). Autophagy and cancer therapy.. Mol. Pharmacol..

[11] Orlikova B., Legrand N., Panning J., Dicato M., Diederich M., Zappia V., Panico S., Russo G.L., Budillon A., Della Ragione F. (2014). Anti-inflammatory and anticancer drugs from nature.. Advances in Nutrition and Cancer. 159..

[12] Yang MH, Liu Y, Kong LY (2016). The innovative drug research base on effective monomer compositions from traditional Chinese medicine.. World Science and Technology/Modernization of Traditional Chinese Medicine and Materia Medica.

[13] Ashford T.P., Porter K.R. (1962). Cytoplasmic components in hepatic cell lysosomes.. J. Cell Biol..

[14] Galluzzi L., Baehrecke E.H., Ballabio A., Boya P., Bravo-San Pedro J.M., Cecconi F., Choi A.M., Chu C.T., Codogno P., Colombo M.I., Cuervo A.M., Debnath J., Deretic V., Dikic I., Eskelinen E.L., Fimia G.M., Fulda S., Gewirtz D.A., Green D.R., Hansen M., Harper J.W., Jäättelä M., Johansen T., Juhasz G., Kimmelman A.C., Kraft C., Ktistakis N.T., Kumar S., Levine B., Lopez-Otin C., Madeo F., Martens S., Martinez J., Melendez A., Mizushima N., Münz C., Murphy L.O., Penninger J.M., Piacentini M., Reggiori F., Rubinsztein D.C., Ryan K.M., Santambrogio L., Scorrano L., Simon A.K., Simon H.U., Simonsen A., Tavernarakis N., Tooze S.A., Yoshimori T., Yuan J., Yue Z., Zhong Q., Kroemer G. (2017). Molecular definitions of autophagy and related processes.. EMBO J..

[15] Towers C.G., Wodetzki D., Thorburn A. (2020). Autophagy and cancer: Modulation of cell death pathways and cancer cell adaptations.. J. Cell Biol..

[16] Onorati A.V., Dyczynski M., Ojha R., Amaravadi R.K. (2018). Targeting autophagy in cancer.. Cancer.

[17] Ma X., Wu Y., Cao G. (2025). S100A8 Mediates autophagy and apoptosis in ovarian cancer cells *via* the PI3K/Akt Pathway.. Discov. Med..

[18] Al-Odat O.S., Guirguis D.A., Schmalbach N.K., Yao G., Budak-Alpdogan T., Jonnalagadda S.C., Pandey M.K. (2022). Autophagy and apoptosis: Current challenges of treatment and drug resistance in multiple myeloma.. Int. J. Mol. Sci..

[19] Gao X., Yin Q., Wang Z. (2025). Olaparib Triggers Mitochondrial Fission Through the CDK5/Drp‐1 Signaling Pathway in Ovarian Cancer Cells.. J. Biochem. Mol. Toxicol..

[20] Ishaq M., Ojha R., Sharma A.P., Singh S.K. (2020). Autophagy in cancer: Recent advances and future directions.. Semin. Cancer Biol..

[21] Amaravadi R.K., Kimmelman A.C., Debnath J. (2019). Targeting autophagy in cancer: Recent advances and future directions.. Cancer Discov..

[22] Zhu J., Zhu H., Zhu Q., Xu S.L., Xiao L., Zhang M.Y., Gao J. (2024). The roles of autophagy, ferroptosis and pyroptosis in the anti-ovarian cancer mechanism of harmine and their crosstalk.. Sci. Rep..

[23] Nokhostin F., Azadehrah M., Azadehrah M. (2022). The multifaced role and therapeutic regulation of autophagy in ovarian cancer.. Clin. Transl. Oncol..

[24] Takeda T., Komatsu M., Chiwaki F., Komatsuzaki R., Nakamura K., Tsuji K., Kobayashi Y., Tominaga E., Ono M., Banno K., Aoki D., Sasaki H. (2019). Upregulation of IGF2R evades lysosomal dysfunction-induced apoptosis of cervical cancer cells *via* transport of cathepsins.. Cell Death Dis..

[25] Ye R., Dai N., He Q., Guo P., Xiang Y., Zhang Q., Hong Z., Zhang Q. (2018). Comprehensive anti-tumor effect of Brusatol through inhibition of cell viability and promotion of apoptosis caused by autophagy *via* the PI3K/Akt/mTOR pathway in hepatocellular carcinoma.. Biomed. Pharmacother..

[26] Meng C.Y., Zhao Z.Q., Bai R., Zhao W., Wang Y.X., Xue H.Q., Sun L., Sun C., Feng W., Guo S.B. (2020). MicroRNA‑22 mediates the cisplatin resistance of osteosarcoma cells by inhibiting autophagy *via* the PI3K/Akt/mTOR pathway.. Oncol. Rep..

[27] Cui Z.Y., Jo E., Jang H.J., Hwang I.H., Lee K.B., Yoo H.S., Park S.J., Jung M.K., Lee Y.W., Jang I.S. (2018). Modified ginseng extract induces apoptosis in HepG2 cancer cells by blocking the CXCL8-mediated akt/nuclear factor-κB signaling pathway.. Am. J. Chin. Med..

[28] Yoshikawa T., Miyamoto M., Aoyama T., Soyama H., Goto T., Hirata J., Suzuki A., Nagaoka I., Tsuda H., Furuya K., Takano M. (2018). JAK2/STAT3 pathway as a therapeutic target in ovarian cancers.. Oncol. Lett..

[29] You L., Wang Z., Li H., Shou J., Jing Z., Xie J., Sui X., Pan H., Han W. (2015). The role of STAT3 in autophagy.. Autophagy.

[30] Lim V., Zhu H., Diao S., Hu L., Hu J. (2019). PKP3 interactions with MAPK-JNK-ERK1/2-mTOR pathway regulates autophagy and invasion in ovarian cancer.. Biochem. Biophys. Res. Commun..

[31] Li X., He S., Ma B. (2020). Autophagy and autophagy-related proteins in cancer.. Mol. Cancer.

[32] Zhu X., Ji M., Han Y., Guo Y., Zhu W., Gao F., Yang X., Zhang C. (2017). PGRMC1-dependent autophagy by hyperoside induces apoptosis and sensitizes ovarian cancer cells to cisplatin treatment.. Int. J. Oncol..

[33] Liu H., Zhou L.L., Wei D.D. (2022). Salidroside down-regulates KRT17 and increases the sensitivity of paclitaxel to chemotherapy in ovarian cancer cells.. J. Tianjin Univ. Tradit. Chin. Med..

[34] Cao W., Li J., Yang K., Cao D. (2021). An overview of autophagy: Mechanism, regulation and research progress.. Bull. Cancer.

[35] Debnath J., Gammoh N., Ryan K.M. (2023). Autophagy and autophagy-related pathways in cancer.. Nat. Rev. Mol. Cell Biol..

[36] Ferro F., Servais S., Besson P., Roger S., Dumas J.F., Brisson L. (2020). Autophagy and mitophagy in cancer metabolic remodelling.. Semin. Cell Dev. Biol..

[37] Hu X., Wang J., Chai J., Yu X., Zhang Y., Feng Y., Qin J., Yu H. (2020). Chaetomugilin J enhances apoptosis in human ovarian cancer A2780 cells induced by cisplatin through inhibiting Pink1/parkin mediated mitophagy.. OncoTargets Ther..

[38] Peng C., Guo Y., Yu Y., Liu F.Y., Han F.J. (2024). Role of autophagy in ovarian cancer based on yin and yang theory.. Zhongguo Shiyan Fangjixue Zazhi.

[39] Singh S.S., Vats S., Chia A.Y.Q., Tan T.Z., Deng S., Ong M.S., Arfuso F., Yap C.T., Goh B.C., Sethi G., Huang R.Y.J., Shen H.M., Manjithaya R., Kumar A.P. (2018). Dual role of autophagy in hallmarks of cancer.. Oncogene.

[40] Kocaturk N.M., Akkoc Y., Kig C., Bayraktar O., Gozuacik D., Kutlu O. (2019). Autophagy as a molecular target for cancer treatment.. Eur. J. Pharm. Sci..

[41] Chang H., Zou Z. (2020). Targeting autophagy to overcome drug resistance: Further developments.. J. Hematol. Oncol..

[42] Bai Z., Peng Y., Ye X., Liu Z., Li Y., Ma L. (2022). Autophagy and cancer treatment: Four functional forms of autophagy and their therapeutic applications.. J. Zhejiang Univ. Sci. B.

[43] Yang P., Song R., Li N., Sun K., Shi F., Liu H., Shen F., Jiang S., Zhang L., Jin Y. (2020). Silica dust exposure induces autophagy in alveolar macrophages through switching Beclin1 affinity from Bcl‐2 to PIK3C3.. Environ. Toxicol..

[44] Mauthe M., Orhon I., Rocchi C., Zhou X., Luhr M., Hijlkema K.J., Coppes R.P., Engedal N., Mari M., Reggiori F. (2018). Chloroquine inhibits autophagic flux by decreasing autophagosome-lysosome fusion.. Autophagy.

[45] Luo D., He F., Liu J., Dong X., Fang M., Liang Y., Chen M., Gui X., Wang W., Zeng L., Fan X., Wu Q. (2024). Pseudolaric acid B suppresses NSCLC progression through the ROS/AMPK/mTOR/autophagy signalling pathway.. Biomed. Pharmacother..

[46] Wang J.H., Wang N., Yuan L.L. (2020). Autophagy of ovarian cancer HO-8910 cells induced by pseudolaric acid-B and its mechanism.. Shiyong Yaowu Yu Linchuang.

[47] Wang H., Luo Y., Chu Z., Ni T., Ou S., Dai X., Zhang X., Liu Y. (2022). Poria Acid, Triterpenoids Extracted from *Poria cocos*, Inhibits the Invasion and Metastasis of Gastric Cancer Cells.. Molecules.

[48] Ma R., Zhang Z., Xu J., Liang X., Zhao Q. (2021). Poricoic acid A induces apoptosis and autophagy in ovarian cancer *via* modulating the mTOR/p70s6k signaling axis.. Braz. J. Med. Biol. Res..

[49] Guo H.M., Wang G., Li N., Sun H., Zhang Y.F. (2020). Antitumor effect and mechanism of Triptolide.. Progress of Anatomical Sciences..

[50] Zhong YY (2021). Study on the mechanism of triptolide regulating JAK2/STAT3/Mcl-1 autophagic pathway against cisplatin-resistant epithelial ovarian cancer.. Nanchang University.

[51] Zhong Y., Le F., Cheng J., Luo C., Zhang X., Wu X., Xu F., Zuo Q., Tan B. (2021). Triptolide inhibits JAK2/STAT3 signaling and induces lethal autophagy through ROS generation in cisplatin‑resistant SKOV3/DDP ovarian cancer cells.. Oncol. Rep..

[52] Zhang X., Xie L., Long J., Xie Q., Zheng Y., Liu K., Li X. (2021). Salidroside: A review of its recent advances in synthetic pathways and pharmacological properties.. Chem. Biol. Interact..

[53] Lu D, Zhang HF, Wang YY (2023). Research progress on antitumor and antiviral pharmacological effects of stephanine.. Guangxi Medicine.

[54] Xiang F.Y., Jiang S.L., Cheng X.Y. (2019). Cepharanthine induces autophagy *via* PI3K / AKT / mTOR signaling path-way in ovarian cancer SKOV3 cells.. Chinese Journal of Pathophysiology..

[55] Liu T., Liu X., Li W. (2016). Tetrandrine, a Chinese plant-derived alkaloid, is a potential candidate for cancer chemotherapy.. Oncotarget.

[56] Shen T.L., Chen M.J., Li Y.Y., Li F. (2020). Tetrandrine induces autophagy of ovarian cancer cells by inhibitingPI3K/Akt/mTOR signaling pathway.. Chinese Journal of Pathophysiology..

[57] Xu J.Y., Guo Y., Yang S., Han F.J. (2021). Study Progress of Effect of Traditional Chinese Medicine Monomer in Intervening Ovarian Cancer by Regulating PI3K/Akt Signaling Pathway.. Zhongguo Shiyan Fangjixue Zazhi.

[58] Zhang X., Hou G., Liu A., Xu H., Guan Y., Wu Y., Deng J., Cao X. (2019). Matrine inhibits the development and progression of ovarian cancer by repressing cancer associated phosphorylation signaling pathways.. Cell Death Dis..

[59] Zhang M.F., Wang J.X., Shen Y.Q. (2019). Research advances of matrine against mammary cancer and ovarian carcinoma.. Yaowu Pingjia Yanjiu.

[60] Zhang L., Li D., Yu S. (2020). Pharmacological effects of harmine and its derivatives: A review.. Arch. Pharm. Res..

[61] Marthandam Asokan S., Mariappan R., Muthusamy S., Velmurugan B.K. (2018). Pharmacological benefits of neferine - A comprehensive review.. Life Sci..

[62] Xu L., Zhang X., Li Y., Lu S., Shan L., Li J., Wang Y., Tian X., Wei J., Shao C., Liu Z. (2016). Neferine induces autophagy of human ovarian cancer cells *via* p38 MAPK/ JNK activation.. Tumour Biol..

[63] Guo X.X., Zhao L.N., Wang J., Liu S., Bi Q.R., Wang Z., Tan N.H. (2018). [Chemical constituents from root barks of Dictamnus dasycarpus and their cytotoxic activities].. Zhongguo Zhongyao Zazhi.

[64] Lv S.H., Liang J.L., Deng M.J., Liu Y.Z., Wang Q. (2023). Dictamnine inhibits proliferation and induces autophage and apoptosis by targeting PI3K /KEAP1 signaling in ovarian cancer cells.. Chin J Oncol Prev Trea..

[65] Zhang Y., Qiu Y.K., Chen J.Y., Bo K.N., Jiang Y.T., Pei Y.H. (2007). Chemical constituents of Bufo bufo gargarizans skin.. J. Shenyang Pharm. Univ..

[66] Gong Q, Liu Y (2020). Research on ROS induced apoptosis and mitophagy stimulated by 19-Hydroxytelocinobufagin (19-HTBG) in ovarian cancer cells.. Zunyi Medical University.

[67] Zhang Y.M., Li T., Xu C.C., Qian J.Y., Guo H., Zhang X., Zhan Z.J., Lu J.J. (2024). Uncover the anticancer potential of lycorine.. Chin. Med..

[68] Hu XM (2017). The research application of endoscopic monitor in the hysteroscope operation; Lycorine induces apoptosis and autophagy, decreases invasion and migration in ovarian cancer and breast cancer.. Lanzhou University, Lanzhou, China.

[69] Rui Z., Chang-Pei X., Jing-Jing Z., Hong-Jun Y. (2020). [Research progress on chemical compositions of Coptidis Rhizoma and pharmacological effects of berberine].. Zhongguo Zhongyao Zazhi.

[70] Li SY, Yu Y, Liu SB (2019). Effect of berberine on endoplasmic reticulum stress-autophagy pathway in human ovarian cancer SKOV3 cells.. Chinese J. Pathophysiol..

[71] Yan X., Yuan C., Wang Z., Xu Z., Wu Z., Wang M., Xu M., Wang Z., Sun Y. (2024). Berberine modulates ovarian cancer autophagy and glycolysis through the LINC01123/P65/MAPK10 signaling axis.. Phytomedicine.

[72] Chen M., Xiao J., El-Seedi H.R., Woźniak K.S., Daglia M., Little P.J., Weng J., Xu S. (2024). Kaempferol and atherosclerosis: From mechanism to medicine.. Crit. Rev. Food Sci. Nutr..

[73] El-Kott A.F., Shati A.A., Al-Kahtani M.A., Alharbi S.A. (2020). Kaempferol induces cell death in A2780 ovarian cancer cells and increases their sensitivity to cisplatin by activation of cytotoxic endoplasmic reticulum-mediated autophagy and inhibition of protein kinase B.. Folia Biol. (Praha).

[74] Ren B., Kwah M.X.Y., Liu C., Ma Z., Shanmugam M.K., Ding L., Xiang X., Ho P.C.L., Wang L., Ong P.S., Goh B.C. (2021). Resveratrol for cancer therapy: Challenges and future perspectives.. Cancer Lett..

[75] Wang H., Peng Y., Wang J., Gu A., Li Q., Mao D., Guo L. (2019). Effect of autophagy on the resveratrol‐induced apoptosis of ovarian cancer SKOV3 cells.. J. Cell. Biochem..

[76] Lang F., Qin Z., Li F., Zhang H., Fang Z., Hao E. (2015). Apoptotic cell death induced by resveratrol is partially mediated by the autophagy pathway in human ovarian cancer cells.. PLoS One.

[77] Cadena-Iñiguez J., Santiago-Osorio E., Sánchez-Flores N., Salazar-Aguilar S., Soto-Hernández R.M., Riviello-Flores M.L., Macías-Zaragoza V.M., Aguiñiga-Sánchez I. (2024). The cancer-protective potential of protocatechuic acid: A narrative review.. Molecules.

[78] Xie Z., Guo Z., Wang Y., Lei J., Yu J. (2018). Protocatechuic acid inhibits the growth of ovarian cancer cells by inducing apoptosis and autophagy.. Phytother. Res..

[79] Lu G., Wang X., Cheng M., Wang S., Ma K. (2023). The multifaceted mechanisms of ellagic acid in the treatment of tumors: State-of-the-art.. Biomed. Pharmacother..

[80] Elsaid F.G., Alshehri M.A., Shati A.A., Al-Kahtani M.A., Alsheri A.S., Massoud E.E., El-kott A.F., El-Mekkawy H.I., Al-Ramlawy A.M., Abdraboh M.E. (2020). The anti‐tumourigenic effect of ellagic acid in SKOV‐3 ovarian cancer cells entails activation of autophagy mediated by inhibiting Akt and activating AMPK.. Clin. Exp. Pharmacol. Physiol..

[81] Tanko A.I., Hosawi S., Moglad E., Afzal M., Ghaboura N., Alzareaa S.I., Osman A., Nadeem M.S., Kazmi I. (2025). Ginsenoside Rg3 in cancer research: Current trends and future prospects - A Review.. Curr. Med. Chem..

[82] Zheng X., Chen W., Hou H., Li J., Li H., Sun X., Zhao L., Li X. (2017). Ginsenoside 20(S)-Rg3 induced autophagy to inhibit migration and invasion of ovarian cancer.. Biomed. Pharmacother..

[83] Jung W.Y., Kim H., Jeon S.J., Park H.J., Choi H.J., Kim N.J., Kim D.H., Jang D.S., Ryu J.H. (2018). Eclalbasaponin II ameliorates the cognitive impairment induced by cholinergic blockade in mice.. Neurochem. Res..

[84] Cho Y.J., Woo J.H., Lee J.S., Jang D.S., Lee K.T., Choi J.H. (2016). Eclalbasaponin II induces autophagic and apoptotic cell death in human ovarian cancer cells.. J. Pharmacol. Sci..

[85] Wang L., Pan G., Tian S., Zhang C., Tao F., Qin J.J., Macranthoside B. (2025). Macranthoside B suppresses the growth of adenocarcinoma of esophagogastric junction by regulating iron homeostasis and ferroptosis through NRF2 inhibition.. Curr. Cancer Drug Targets.

[86] Shan Y., Guan F., Zhao X., Wang M., Chen Y., Wang Q., Feng X. (2016). Macranthoside B induces apoptosis and autophagy *via* reactive oxygen species accumulation in human ovarian cancer A2780 cells.. Nutr. Cancer.

[87] Tian Y., Gong G.Y., Ma L.L., Wang Z.Q., Song D., Fang M.Y. (2020). Anti-cancer effects of Polyphyllin I: An update in 5 years.. Chem. Biol. Interact..

[88] Chen DW, Huang JZ (2022). Inhibitory effect and preliminary mechanism of chinese herbal monomelic polyphyllin I On Ovarian Cancer Cell SKOV3 and ID8.. Guangdong Medical University.

[89] Wu ZY, Huang JZ (2022). PolyphyllinⅠ on Ishikawa and Skov3 cells by autophagy.. Guangdong Medical University.

[90] Zhu Y., Lai Y. (2023). Pharmacological properties and derivatives of saikosaponins—a review of recent studies.. J. Pharm. Pharmacol..

[91] Liu L., Zhang L.F., Fang Z.N., Wang X.Y. (2017). Study on the apoptosis of ovarian cancer SKOV 3 cells induced by saikosaponin.. China Medical Herald..

[92] Zhang L., Liu Y., Lei X., Liu X., Sun H., Liu S. (2023). Astragaloside II enhanced sensitivity of ovarian cancer cells to cisplatin *via* triggering apoptosis and autophagy.. Cell Biol. Int..

[93] Singh D., Singh R. (2025). Pharmacological and Therapeutic Potential of a Natural Flavonoid Icariside II in Human Complication.. Curr. Drug Targets.

[94] Yuan D., Guo T., Qian H., Ge H., Zhao Y., Huang A., Wang X., Cao X., Zhu D., He C., Yu H. (2022). Icariside II suppresses the tumorigenesis and development of ovarian cancer by regulating miR‐144‐3p/IGF2R axis.. Drug Dev. Res..

[95] Deng J., Wu T.F., Ye M.Q., Ma Z.G., Cao H., Zhang Y. (2023). A Comparative Identification Study on Alpiniae katsumadai Semen and Alpiniae henryi Semen.. Strait Pharmacy..

[96] Shi D., Zhao D., Niu P., Zhu Y., Zhou J., Chen H. (2018). Glycolysis inhibition *via* mTOR suppression is a key step in cardamonin-induced autophagy in SKOV3 cells.. BMC Complement. Altern. Med..

[97] Shi D., Niu P., Heng X., Chen L., Zhu Y., Zhou J. (2018). Autophagy induced by cardamonin is associated with mTORC1 inhibition in SKOV3 cells.. Pharmacol. Rep..

[98] Ma N., Li Y.J., Fan J.P. (2018). Research Progress on Pharmacological Action of Diosmetin.. Journal of Liaoning University of TCM..

[99] Zhang FJ, Zhang SL (2021). Effects and mechanisms of diosmetin on proliferation and apoptosis in ovarian cancer cells.. Jilin University.

[100] Zhang RJ, Yuan MM (2021). Studies of the anti-tumor effect and mechanism of nobiletin on ovarian cancer.. Southern Medical University.

[101] Jiang Y.P., Guo H., Wang X.B. (2018). Nobiletin (NOB) suppresses autophagic degradation *via* over-expressing AKT pathway and enhances apoptosis in multidrug-resistant SKOV3/TAX ovarian cancer cells.. Biomed. Pharmacother..

[102] Li R., Zheng C., Shiu P.H.T., Rangsinth P., Wang W., Kwan Y.W., Wong E.S.W., Zhang Y., Li J., Leung G.P.H. (2023). Garcinone E triggers apoptosis and cell cycle arrest in human colorectal cancer cells by mediating a reactive oxygen species–dependent JNK signaling pathway.. Biomed. Pharmacother..

[103] Xu X.H., Chen Y.C., Xu Y.L., Feng Z.L., Liu Q.Y., Guo X., Lin L-G., Lu J-J. (2021). Garcinone E blocks autophagy through lysosomal functional destruction in ovarian cancer cells.. World J. Tradit. Chin. Med..

[104] Zhang Y., Ding X., Zhang Q., Zeng C., Chen H., Lu L. (2024). Trichosanthin elicits antitumor activity *via* MICU3 mediated mitochondria calcium influx.. J. Adv. Res..

[105] Cao C., Qi H., Chen F., He J. (2017). Trichosanthin inhibits human ovarian cancer cells growth due to apoptosis and autophagy.. Int. J. Clin. Exp. Med..

[106] Zhao T.T., Xu Y.Q., Hu H.M., Gong H.B., Zhu H.L. (2019). Isoliquiritigenin (ISL) and its Formulations: Potential Antitumor Agents.. Curr. Med. Chem..

[107] Chen H.Y., Huang T.C., Shieh T.M., Wu C.H., Lin L.C., Hsia S.M. (2017). Isoliquiritigenin induces autophagy and inhibits ovarian cancer cell growth.. Int. J. Mol. Sci..

[108] Kamatou G.P.P., Vermaak I., Viljoen A.M., Lawrence B.M. (2013). Menthol: A simple monoterpene with remarkable biological properties.. Phytochemistry.

[109] Yu Y, Liu SB, Li SY, Xu L, Xu Y (2017). Induction effect of myricetin on autophagy in skov3 cells and promoting effect on mitochondrial fission.. J. Jilin University.

[110] Wang Q., Wei H.C., Zhou S.J., Li Y., Zheng T.T., Zhou C.Z., Wan X.H. (2022). Hyperoside: A review on its sources, biological activities, and molecular mechanisms.. Phytother. Res..

[111] Wen Y., Wang Y., Zhao C., Zhao B., Wang J. (2023). The Pharmacological Efficacy of Baicalin in Inflammatory Diseases.. Int. J. Mol. Sci..

[112] Choi B.Y., Joo J.C., Lee Y.K., Jang I.S., Park S.J., Park Y.J. (2017). Anti-cancer effect of Scutellaria baicalensis in combination with cisplatin in human ovarian cancer cell.. BMC Complement. Altern. Med..

[113] Mahwish I.M., Imran M., Naeem H., Hussain M., Alsagaby S.A., Al Abdulmonem W., Mujtaba A., Abdelgawad M.A., Ghoneim M.M., El-Ghorab A.H., Selim S., Al Jaouni S.K., Mostafa E.M., Yehuala T.F. (2025). Antioxidative and Anticancer Potential of Luteolin: A Comprehensive Approach Against Wide Range of Human Malignancies.. Food Sci. Nutr..

[114] Xu R.M., Cao Y.D., Lian X.F., Cai Y., Shi S.J., Zhang S. (2024). A2780 cells luteolin-induced apoptosis and autophagy in human ovarian cancer *via* AKT-mTOR pathway.. Hainan Yixueyuan Xuebao.

[115] Liu Q., Zhu D., Hao B., Zhang Z., Tian Y. (2018). Luteolin promotes the sensitivity of cisplatin in ovarian cancer by decreasing PRPA1-medicated autophagy.. Cell. Mol. Biol..

[116] Zhou A., Peng N., Yang L., Yang S., Wang J. (2025). Tanshinone regulated gut microbiota and TMAO to improve high-fat diet induced atherosclerosis in APOE^−/−^ mice.. BMC Microbiol..

[117] Li WY, Feng C, Ren CH, Mao YC, Zhang ZL, Feng H (2023). Effects of Tanshinone IIA on proliferation, migration and autophagy of human ovarian cancer A2780 cells.. Journal of Mudanjiang Medical University.

[118] Zhang H.L., Luo X.P. (2021). Research Progress on the Biological Activity of Juglone, the Main Component of Walnut Green Peel.. Med. Chem. Res..

[119] Zhang J., Ma L.J., Wang Y.H. (2019). The effect and mechanism of juglone on autophagy level in human ovarian cancer cell line SKOV3.. Zhongguo Yaowu Yu Linchuang.

[120] García-Vilas J.A., Quesada A.R., Medina M.A. (2015). Damnacanthal, a noni anthraquinone, inhibits c-Met and is a potent antitumor compound against Hep G2 human hepatocellular carcinoma cells.. Sci. Rep..

[121] Li R., Li H., Lan J., Yang D., Lin X., Xu H., Han B., Yang M., Su B., Liu F., Jiang W. (2022). Damnacanthal isolated from morinda species inhibited ovarian cancer cell proliferation and migration through activating autophagy.. Phytomedicine.

[122] Qi N., Duan W.J., Li Y.J., Kang S.M., Zhang S.Q., Zhou X.T. (2020). Research Progress on the Pharmacological Action of Muscone.. Modernization of Traditional Chinese Medicine and Materia Materia-World Science and Technology..

[123] Pan Y., Zhou J., Zhang W., Yan L., Lu M., Dai Y., Zhou H., Zhang S., Yang J. (2021). The Sonic Hedgehog signaling pathway regulates autophagy and migration in ovarian cancer.. Cancer Med..

[124] Wang A.H., Zhang F.Z., Wang H.Y. (2024). Impacts of muscone on malignant progression of ovarian cancer cells by regulating SHH mediated autophagy.. Tianjin Yi Yao.

[125] Zhang X., Chen L.X., Ouyang L., Cheng Y., Liu B. (2012). Plant natural compounds: Targeting pathways of autophagy as anti‐cancer therapeutic agents.. Cell Prolif..

[126] Ning Y, Fu YL, Zhang QH, Zhang C, Chen Y (2019). Inhibition of *in vitro and in vivo* ovarian cancer cell growth by pinoresinol occurs by way of inducing autophagy, inhibition of cell invasion, loss of mitochondrial membrane potential and inhibition ras/MEK/ERK signalling pathway.. Journal of BUON: Official journal of the Balkan Union of Oncology.

[127] Singh G., Singh M.K. (2023). An overview on sources, biosynthesis and bioactivities of osthole: APotential bioactive compound.. Curr. Bioact. Compd..

[128] Liang J., Zhou J., Xu Y., Huang X., Wang X., Huang W., Li H. (2020). Osthole inhibits ovarian carcinoma cells through LC3-mediated autophagy and GSDME-dependent pyroptosis except for apoptosis.. Eur. J. Pharmacol..

[129] Guan H.T., Yu C.H. (2024). Research Progress on Anti -tumor Mechanism of Curcumin.. Zhonghua Zhongyiyao Xuekan.

[130] Adki K.M., Kulkarni Y.A. (2020). Chemistry, pharmacokinetics, pharmacology and recent novel drug delivery systems of paeonol.. Life Sci..

[131] Liu L., Pang Y., Zhao X., Li R., Jin C., Xue J., Dong R., Liu P. (2019). Curcumin induces apoptotic cell death and protective autophagy by inhibiting AKT/mTOR/p70S6K pathway in human ovarian cancer cells.. Arch. Gynecol. Obstet..

[132] Gao L., Wang Z., Lu D., Huang J., Liu J., Hong L. (2019). Paeonol induces cytoprotective autophagy *via* blocking the Akt/mTOR pathway in ovarian cancer cells.. Cell Death Dis..

[133] Zhang Z (2018). Correlation of autophagy and AKT / mTOR signaling pathway in the reversal of carboplatin resistance in ovarian cancer cells by curcumin.. China Medical University.

[134] Lu Q., Luo S., Shi Z., Yu M., Guo W., Li C. (2022). Nitidine chloride, a benzophenanthridine alkaloid from *Zanthoxylum nitidum* (Roxb.) DC., exerts multiple beneficial properties, especially in tumors and inflammation-related diseases.. Front. Pharmacol..

[135] Feng F., Zhang J., Lian C., Huang Y., Hu P., Cao Y., Zhang Z. (2023). Nitidine Chloride Triggers Autophagy and Apoptosis of Ovarian Cancer Cells through Akt/mTOR Signaling Pathway.. Curr. Pharm. Des..

[136] Barras B.J., Ling T., Rivas F. (2024). Recent Advances in Chemistry and Antioxidant/Anticancer Biology of Monoterpene and Meroterpenoid Natural Product.. Molecules.

[137] Shi Y.C., He Y., Yang Y., Fan Y.J., Yang F., Zhan L. (2022). Chrysin inhibits proliferation and promotes apoptosis of ovarian cancer cells by inducing autophagy.. Acta Universitatis Medicinalis Anhui..

[138] Atolani O., Usman M.A., Adejumo J.O., Ayeni A.E., Ibukun O.J., Kola-Mustapha A.T., Njinga N.S., Quadri L.A., Ajani E.O., Amusa T.O., Bakare-Odunola M.T., Oladiji A.T., Alqahtani A., Abbas M., Kambizi L. (2024). Isolation, characterization and anti-inflammatory activity of compounds from the *Vernonia amygdalina.*. Heliyon.

[139] Tu J.H., Lou W.H., Huang R., Zhao A.M. (2015). Vernolepin regulates apoptosis and autophagy *via* microtubule formation in ovarian carcinoma cells.. Bangladesh J. Pharmacol..

[140] Zhang L, Wu J, Xue J, Jia Z, Zhou X, Hu G (2025). Investigating the Therapeutic Potential and Molecular Mechanisms of Daphnetin: A Comprehensive Review.. Recent Patents on Anticancer Drug Discovery.

[141] Fan X., Xie M., Zhao F., Li J., Fan C., Zheng H., Wei Z., Ci X., Zhang S. (2021). Daphnetin triggers ROS-induced cell death and induces cytoprotective autophagy by modulating the AMPK/Akt/mTOR pathway in ovarian cancer.. Phytomedicine.

[142] Tuli H.S., Aggarwal V., Kaur J., Aggarwal D., Parashar G., Parashar N.C., Tuorkey M., Kaur G., Savla R., Sak K., Kumar M. (2020). Baicalein: A metabolite with promising antineoplastic activity.. Life Sci..

[143] Wang Y.F., Xu Y.L., Tang Z.H., Li T., Zhang L.L., Chen X., Lu J.H., Leung C.H., Ma D.L., Qiang W.A., Wang Y.T., Lu J.J. (2017). Baicalein induces beclin 1-and extracellular signal-regulated kinase-dependent autophagy in ovarian cancer cells.. Am. J. Chin. Med..

[144] Yang D., Wang T., Long M., Li P. (2020). Quercetin: Its Main Pharmacological Activity and Potential Application in Clinical Medicine.. Oxid. Med. Cell. Longev..

[145] Liu Y., Gong W., Yang Z.Y., Zhou X.S., Gong C., Zhang T.R., Wei X., Ma D., Ye F., Gao Q.L. (2017). Quercetin induces protective autophagy and apoptosis through ER stress *via* the p-STAT3/Bcl-2 axis in ovarian cancer.. Apoptosis.

[146] Naz I., Ramchandani S., Khan M.R., Yang M.H., Ahn K.S. (2020). Anticancer potential of raddeanin a, a natural triterpenoid isolated from anemone raddeana regel.. Molecules.

[147] Zhao F., Gao Y., Chu X., Chen J., Huang L., Zhao J., Zhang J., Zhao S. (2017). ROS attenuates the antitumor effect of Raddeanin on ovarian cancer cells Skov3.. Int. J. Clin. Exp. Pathol..

[148] Chu XM (2015). The effect and mechanism of ROS in the inhibition of proliferation of Raddeanin A on ovarian cancer cell Skov3.. Jilin University.

[149] Bukowski K., Kciuk M., Kontek R. (2020). Mechanisms of Multidrug Resistance in Cancer Chemotherapy.. Int. J. Mol. Sci..

[150] Li S.H., Chen Y.H., Lv Y. (2022). Research progress on autophagy and chemotherapy resistance of cancer.. Anti-tumor Pharmacy..

[151] Capasso L., De Masi L., Sirignano C., Maresca V., Basile A., Nebbioso A., Rigano D., Bontempo P. (2025). Epigallocatechin Gallate (EGCG): Pharmacological Properties, Biological Activities and Therapeutic Potential.. Molecules.

[152] Wang Y., Yin Z.Y., Liu T., Zheng B.B., Zhang J.B., Li Y.Y. (2016). Effects of EGCG on the autophagy of human ovarian cancer SKOV3 cells.. Xuzhou Medical University..

[153] Teng C., Chen J.W., Shen L.S., Chen S., Chen G.Q. (2025). Research advances in natural sesquiterpene lactones: Overcoming cancer drug resistance through modulation of key signaling pathways.. Cancer Drug Resist..

[154] Xie Z (2019). Study on the mechanism of scutellarin and isoalantolactone in sensitizing ovarian cancer cells to cisplatin.. Wu Han University.

[155] Liang X (2023). Inhibition of STX17–SNAP29–VAMP8 complex formation by costunolide sensitizes ovarian cancer cells to cisplatin *via* the AMPK/mTOR signaling pathway.. North Sichuan Medical College.

[156] Wang Y, Wu X, Liu FJ, Zhang CL (2024). Effects of magnoflorine on the migration, invasion and stemness characteristics of lung cancer cells by regulating the CD44s/STAT3 pathway.. Shaanxi Medical Journal.

[157] Zhang Y., He X., Fan L., Zhang Q. (2024). Magnoflorine inhibits cisplatin-induced protective autophagy by down-regulating HMGB1 and increases drug sensitivity in drug-resistant ovarian cancer cells.. Eur. J. Gynaecol. Oncol..

[158] Kaur K., Sharma R., Singh A., Attri S., Arora S., Kaur S., Bedi N. (2022). Pharmacological and analytical aspects of alkannin/shikonin and their derivatives: An update from 2008 to 2022.. Chin. Herb. Med..

[159] Fang K., Qiu F., Ding Y.N., Deng D.N., Tan Q.F. (2023). Effects of Shikonin on Ovarian Cancer SK- OV- 3 Cells Through Autophagy Mediated by Keap1/Nrf2Signaling Pathway.. Zhongyao Xinyao Yu Linchuang Yaoli.

[160] Wang S., Li J., Xu S., Wang N., Pan B., Yang B., Zheng Y., Zhang J., Peng F., Peng C., Wang Z. (2024). Baohuoside I chemosensitises breast cancer to paclitaxel by suppressing extracellular vesicle/CXCL1 signal released from apoptotic cells.. J. Extracell. Vesicles.

[161] Zhou Y., Liu T., Wu Q., Wang H., Sun Y. (2023). Baohuoside I inhibits resistance to cisplatin in ovarian cancer cells by suppressing autophagy *via* downregulating HIF‐1α/ATG5 axis.. Mol. Carcinog..

[162] Cai J., Wen H., Zhou H., Zhang D., Lan D., Liu S., Li C., Dai X., Song T., Wang X., He Y., He Z., Tan J., Zhang J. (2023). Naringenin: A flavanone with anti-inflammatory and anti-infective properties.. Biomed. Pharmacother..

[163] Zhu J., Lin S., Zou X., Chen X., Liu Y., Yang X., Gao J., Zhu H. (2023). Mechanisms of autophagy and endoplasmic reticulum stress in the reversal of platinum resistance of epithelial ovarian cancer cells by naringin.. Mol. Biol. Rep..

[164] Wang Y., Liu M., Jia X., Yang Q., Du Y. (2025). AMPK/mTOR/ULK1 pathway participates in autophagy induction by curcumin in colorectal adenoma mouse model.. Drug Dev. Res..

[165] Dai L.B., Zhong J.T., Shen L.F., Zhou S.H., Lu Z.J., Bao Y.Y., Fan J. (2021). Radiosensitizing effects of curcumin alone or combined with GLUT1 siRNA on laryngeal carcinoma cells through AMPK pathway‐induced autophagy.. J. Cell. Mol. Med..

[166] Kang H.S., Lim H.K., Jang W.Y., Cho J.Y. (2025). Anti-Colorectal Cancer Activity of *Panax* and Its Active Components, Ginsenosides: A Review.. Int. J. Mol. Sci..

[167] Wang L., Zhang Y., Song Z., Liu Q., Fan D., Song X. (2023). Ginsenosides: A potential natural medicine to protect the lungs from lung cancer and inflammatory lung disease.. Food Funct..

[168] Xu J., Hu M., Li Y., Gong H., Zhang X., He Z., Xiao C., Yang C., Zeng J. (2025). Berberine Enhances the Sensitivity of Colorectal Cancer Cells to 5‐FU Through Smoothing Endoplasmic Reticulum Stress‐Mediated Autophagic Flux.. Cell Biol. Int..

[169] Ramesh G., Das S., Bola Sadashiva S.R. (2020). Berberine, a natural alkaloid sensitizes human hepatocarcinoma to ionizing radiation by blocking autophagy and cell cycle arrest resulting in senescence.. J. Pharm. Pharmacol..

[170] Wei P., Zhang X., Yan C., Sun S., Chen Z., Lin F. (2025). Mitochondrial dysfunction and aging: Multidimensional mechanisms and therapeutic strategies.. Biogerontology.

[171] Kovale L., Singh M.K., Kim J., Ha J. (2024). Role of autophagy and ampk in cancer stem cells: Therapeutic opportunities and obstacles in cancer.. Int. J. Mol. Sci..

[172] Baird L., Yamamoto M. (2020). The molecular mechanisms regulating the KEAP1-NRF2 Pathway.. Mol. Cell. Biol..

[173] Galluzzi L., Pietrocola F., Bravo-San Pedro J.M., Amaravadi R.K., Baehrecke E.H., Cecconi F., Codogno P., Debnath J., Gewirtz D.A., Karantza V., Kimmelman A., Kumar S., Levine B., Maiuri M.C., Martin S.J., Penninger J., Piacentini M., Rubinsztein D.C., Simon H.U., Simonsen A., Thorburn A.M., Velasco G., Ryan K.M., Kroemer G. (2015). Autophagy in malignant transformation and cancer progression.. EMBO J..

[174] Patra S., Mishra S.R., Behera B.P., Mahapatra K.K., Panigrahi D.P., Bhol C.S., Praharaj P.P., Sethi G., Patra S.K., Bhutia S.K. (2022). Autophagy-modulating phytochemicals in cancer therapeutics: Current evidences and future perspectives.. Semin. Cancer Biol..

[175] Lei L., Zhang J., Wei R., Dong B., Wang X., Zhou Y. (2025). Deciphering the dual role of autophagy in gastric cancer and gastroesophageal junction cancer: From tumor suppression to cancer progression.. Discover Oncology.

[176] Murugan S., Amaravadi R.K. (2016). Methods for Studying Autophagy Within the Tumor Microenvironment.. Adv. Exp. Med. Biol..

[177] Ma W., Lu Y., Jin X., Lin N., Zhang L., Song Y. (2024). Targeting selective autophagy and beyond: From underlying mechanisms to potential therapies.. J. Adv. Res..

